# Supplementary material for: The role of the physical environment in stroke recovery: Evidence-based design principles from a mixed-methods multiple case study
Source: PLoS One. 2023 Jun 9;18(6):e0280690. doi: 10.1371/journal.pone.0280690 (PMC10256226; doi:10.1371/journal.pone.0280690)
Supplement: S1 Table — (DOCX) [file pone.0280690.s001.docx]

**S1 Table. Description of the environments of the two cases in the ENVIRONS Study**.

|  | Case 1 (St George’s Hospital) | Case 2 (Bendigo Hospital) | Comparison^a^ |
| --- | --- | --- | --- |
| Remoteness | Metropolitan (in a suburb of Melbourne) | Inner regional (Bendigo, rural town) | Different |
| Funding | Public | Public | Similar |
| No. of  buildings | Rehabilitation facility is housed in one building. Some  additional services (e.g., a transitional care facility) are housed in a separate building. | The majority of rehabilitation services are in the main hospital building, which is on a large campus (see row below).  Hydrotherapy and some rehabilitation staff offices are in a separate building on the same campus. | Similar |
| Local context | On a main street, residential area. Tram stop directly  outside building, bus stop on corner. Plentiful paid visitor car park and free on-street parking. Active construction site behind building. | On a hill overlooking town and surrounding countryside. At the intersection of two main roads, bus stop is a short walk. Limited paid visitor car park and some free on-street parking. Hospital campus is large and includes a university campus, cafes, and  landscaped outdoor areas. | Different |
| Type of rehab ward | One rehabilitation ward (orthopaedic and neurological). | One rehabilitation ward (neurological and GEM). There is also a musculoskeletal rehab ward not included in this case study. | Different |
| No. of beds | 31 beds on the rehabilitation ward (but funded for 30). | 32 beds on the neurological rehabilitation ward (some of which are dedicated to GEM patients). | Similar |
| Nurse to  patient ratio | 1:5 during the day | 1:5 during the day | Similar |
| Free-Standing | Free-standing facility, not attached to a tertiary hospital. | Part of a large tertiary hospital which services the region. | Different |
| Year built | The oldest part of the building was built prior to 1912  (entrance), and the newest in 2009 (rehabilitation gym). | Main hospital building built 2017. | Different |
| Renovations | The building has been renovated multiple times with many extensions added over the years. | None | Different |
| Purpose-built status | Most of the building (including the ward) was not purpose-built for rehabilitation, but the gym was. | Rehabilitation ward was designed following same template as all other wards, not purpose-built. | Similar |
| Original  purpose | Medical, various | Medical, unspecified | Similar |
| Floors | Rehabilitation services span two floors (ward on the 1^st^ floor ward and therapy areas on the ground floor) | Rehabilitation ward and gym are on the 6^th^ floor, which is the top floor of the building. | Different |
| Ward layout | Rectangular ‘racetrack’ with patient’s rooms on the outside and staff areas (kitchen, medication storage) inside the  rectangle. No dining area; meals eaten in bedrooms. | Triangular ‘racetrack’ with patient’s rooms on the outside and staff areas (kitchen, medication storage, offices, meeting room)  inside the triangle. No dining area; meals eaten in bedrooms. | Similar |
| Ward  atmosphere | Hallways feel narrow. Equipment is often stored in  hallways or bathrooms. Posters and art in hallways, less  colour in bedrooms. Patients bring in personal items, stick things on walls of their bedrooms. No staff area on the ward, except the nurses’ station and offices. Patients not  allowed off-ward without permission. Busy atmosphere. | Feels big, new, clean, white. Walls are bare, except for some  notices and a tree-like freeze along hallway. Hallways are wide, long and light-filled. There is a staff hand-over room, a reception desk, and 2 nurses’ stations. Patients not allowed off-ward  without permission. Those who are given permission are issued a ‘green card’. Ward is busy in some parts, quiet in others. | Different |
| Therapy areas | Gym, OT kitchen, and OT therapy room are on ground floor. Speech therapy rooms are on the ward (1^st^ floor). The gym is new and very large with big windows. Therapy  garden on ground floor (open access). No hydrotherapy. | Gym and a mixed-use therapy room on same floor as ward. Gym is small. Hydrotherapy and another gym in separate building. No OT kitchen, but ‘community room’ with a kitchen that patients can use with staff. Therapy garden on floor below (no access  without staff). Environmental enrichment station on the ward. | Different |
| Communal  areas | Patient lounge (‘sunroom’) on ward. Sunroom is narrow with windows along one side, a cushioned bench, two chairs, a small table, TV, and books/magazines. Tea making facilities in sunroom, but no water source. Waiting area on the ground floor is occasionally used as a space to sit and talk. Café on ground floor. | Patient lounge and ‘lounge nooks’ on ward. Patient lounge has central table with chairs, TV, books/magazines, iPads. The lounge nooks each have 2-3 chairs placed near a window at the end of a hallway. Tea making facilities in hallway. Four food outlets/cafés on lower floors, and another in a separate building. | Different |
| Outdoor areas | Café outdoor seating and therapy garden on ground floor (next to carpark). Benches out the front of the hospital (next to another car park). Mature native trees at front and sides of building. Balcony off the sunroom is kept locked. | Large, landscaped outdoor areas either side of the hospital on the ground floor. Therapy garden on 4^th^ floor (no access without staff). Balcony for staff on the same floor as rehab ward (not accessible to patients). Balcony next to gym is kept locked. | Different, but similarly limited and all off-ward |
| Bedrooms | 16 single-bed rooms; 1 three-bed room; 3 four-bed rooms. All have shared bathrooms, except 7 of the single-bed rooms which have ensuite. These 7 single-bed rooms are large, but the ones without ensuite are quite small. One of the single-bed rooms includes kitchenette (but no ensuite). | There are 16 single-bed rooms; 6 two-bed rooms; and 1 four-bed room. All of the single-bed rooms have an ensuite, as do the two-bed rooms. The four-bed room has 2 ensuites. Most bedrooms are large, with high ceilings, and big windows. | Different |
| % single-bed rooms | 52% of the beds are in single-bed rooms. | 50% of the beds are in single-bed rooms | Similar |
| Visiting hrs | 8am to 8pm, 7 days per week | 3pm-8pm Mon to Fri, 11am-8pm weekends, often not enforced | Similar |

OT = Occupational Therapy; rehab = rehabilitation

^a^Similarities and differences between the cases were judged based on observations and field notes.
