# Supplementary material for: The role of the physical environment in stroke recovery: Evidence-based design principles from a mixed-methods multiple case study
Source: PLoS One. 2023 Jun 9;18(6):e0280690. doi: 10.1371/journal.pone.0280690 (PMC10256226; doi:10.1371/journal.pone.0280690)
Supplement: S2 Table — Minimum data set for the qualitative data from the ENVIRONS study. (DOCX) [file pone.0280690.s002.docx]

## S2 Table. Illustrative quotes from the walk-through semi-structured interviews.

## Minimum data set for the qualitative data from the ENVIRONS study.

**Theme 1 – Entrapment & escape**

| **Sub-theme** | **Case 1 quotes** | **Case 2 quotes** |
| --- | --- | --- |
| **1.1. The environment is restrictive and boring** | 1.1.20 ““Q: What do you think they could change in the building to help you get out of your room more? A: Put more exit doors.” 2656-01  1.3.28 “I’m bored witless and that’s why I keep doing [crosswords]. That’s about] getting my memory back. So I’m doing all of that and I’m going to the gym and all that cos for me to just sit somewhere for a … Yeah but for me for me it’s it’s boring. But that’s just because I’m I don’t stay at home at home you know I’m it very rare that I wouldn’t go out during the day. It might be just to walk to down get a bottle of milk or carton of milk, but I wouldn’t take the car, and I’ve got this shopping centre, and then cos I’ve got a post office box then I’d walk over about oh it’d be a couple of k over, so about 4 k over and back um and to get my mail, and I’ll have a coffee and yeah. And then I run into people and we have coffee you know regularly you know. Just doing things. And here when I wasn’t even allowed downstairs.” 2656-02  “[My bedroom] stinks … because I’m sitting here all day … It’s not good for the brain. It’s not good for you. You’re talking to nobody. … I don’t like anything in the room because it’s still locking you up." 2656-08  1.1.7. “Like everybody that would be here there’d be only one thought in their head just to get out and so to get better and get out. It’s a good place to be when you’re sick but I think when you’re getting well you don’t wanna be here. You know. You wanna be out.” 2656-08  “I usually stay [in my bedroom] because there’s nothing interesting out there so not really keen to look at, everything is the same. Yeah so that’s why I stay in this room” 2656-09  1.1.9. “like a holiday camp. With nothing to do.” 2656-10  1.1.5. “[I] set the [bed] alarm off every time I moved, which was very annoying. It woke me up. I was trying to get a comfortable position in bed and this silly machine wouldn’t let me. The slightest movement and the staff eruption to see that I’m still in bed. [They] reset the machine and then if I moved off it would go again. So that happened five or six times a night.” 2656-10  “[In] this room I feel a bit like I’m stir crazy” 2656-18  1.1.15 “While I’m in this room? I get frightfully bored. Yes what I do is have my meals here, go to the toilet, do my exercises that I’m supposed to do, read the paper, and wait for another day to go by" 2656-19 | 1.1.18 “you get a bit edgy just looking at four walls most of the day” (2657-10)  1.1.11 “it’s all the same when you’re in here” (2657-04)  1.1.2.“You know just seeing people over and over and over and over and hearing the same things over and over and over and over oh. Just gives you the shits. … where else is there to bloody go!? Nowhere, is there? That’s what I mean. You’re too enclosed in the place. … At least once or twice a week if I could walk outside here you know just … wouldn’t make me feel as if I’m enclosed … you got to be able to move around the place outside occasionally.” (2657-15)  “This room doesn’t [make me feel motivated]. The staff does. Mm. They’re very good. and they’ve been very encouraging too … I think it’s the people that motivate you most. … [The ward] doesn’t, what’s the word, innovative or whatever the right word is, it doesn’t encourage me to want to do anything. You know if there was a lot of books then I could go and see what there was to read” 2657-09  2.2.18 “that’s when I find that you can get bored. Is when you can’t just ah get up and go to the toilet or get up and go for a walk. You’ve gotta ring the bell and someone’s gotta be available to take you where you want to go [yeah]. That’s the only time I find that you could get bored.” 2657-06  1.1.16 “every day falls into the next one, every day’s Friday yeah it’s as if I’ve been on holidays since I’ve been in. Every day folds into the next one, you know. You don’t get excited cos it’s Friday cos you know you’ve got the weekend coming up. … it’s important to have a sense of anticipation for certain things like the weekend and that but I know I can’t get out so I’m stumped you know.” 2657-04  1.1.19 “There was a lady in this half and she kept this blind closed all the time (laughs). I didn’t see any outdoors. [I felt] sort of closed in you know. Yeah very small and boxy. Small and boxy. Because it’s not really a big area is it? This room’s is a small room isn’t it? At least you got the passage there and you can see people coming and going you know. … you like to know you’re not the only one here (laughs). Don’t want to feel isolated from the world do you? … Just people coming in and saying hello [help me feel connected with the world] .. and here’s your tablet or do you want anything or how are you and everything.” 2657-02  “I find the [bed]rooms are very good. They’re quite roomy … one lady I had thought the rooms were too small, but I don’t find that. I find they’re big and nice … I find this building is very spacious. You don’t feel as if you’re in a hospital environment really. … I find there’s plenty of cupboard space, wardrobe space, draw space um and quite it’s quite roomy to get around. … [and] I think [the hallways are] lovely. They don’t close you in. You know? And you can walk side by side and people can walk past you.” 2657-06  1.2.27 “deep down we really hate the place, everyone does. You just wanna get out of here but you also know that you’ve got to get better before we can.” 2657-14 |
| **1.2. Connection, purpose, and change are means of escape** | 1.2.2. “[I spend a lot of time looking out the bed room window] cos there’s construction going on. It’s changing and it keeps me head going. … So that’s progress you know, you can see what they’re doing. I call that my TV wall! [laughs] … Keeps me head level. You know I just go there [to the window] in the morning and see what’s going on … You just see blokes running around like ants. They’re all doing something. That keeps my head in a good position. I think if I didn’t have that I’d be a little bit more wound up. I could sit there for hours.” 2656-01  1.2.4. “I’ve got the bed that’s right hear near the window or whatever, but eh makes you feel eh that you are not alone lonely sort of thing you know you look out the window and you see the odd cars going past and and the feeling is you go ah I’m not alone, you know there’s a bit of traffic going through here, there’s some people there going through here. You know that sort of thing.” 2656-04  1.2.8. “there’s the room with desks and um and I’ve changed those the they they couldn’t put the chairs underneath the um chairs so I chucked those and put that can get right underneath and it’s great … so but even if you even you aiming that way or just slight that way it can be better if it’s like that or that way is not exact as best or something you want to be because you want something of interest or something like likable something that you want yeah so R: Do you mean the position of the chair? M: Yes because yes [because if I just sit] anywhere but then I want to look at that the ta ta to me the window. But then it depends on where the blinds are going and then the sun that coming a certain way, but if you look that way, that’s like that’s ok but then if you turn that way it’s better because that way I can see oh there are tr oh cars there they can see people are walking they can see bicycles, yeah but I wouldn’t have been able to seen that but if I had have looked that way or so just things are to me are like interest are observance observance to just things that anything happening it’s like” 2656-11  1.3.2. “There’s good views from [the gym] … being able to see off into the distance is wonderful. … another world. Magic. … Yeah it’s freedom, like when you’re locked in a room all the time it’s lovely to see the distance, the distance and the hills, even though they’re covered in smog. And it’s the freedom of the big wide open space.” 2656-10  1.2.11. “I look out that window all day. I look out that window all day especially when it’s raining. I look out that window and if they pull the blinds down I get ‘em to pull them up. … Well I had that blind up and this um and this blind up so I can look out of both. You know, there’s nothing else [to look at]. Nothing else. Just the trees. This tree here wasn’t as bushy about three or four weeks ago you could see right through. Now you can’t cos it’s all bushy.” 2656-08  1.2.12. “[The view from the] window outside it’s good. Nice view. The same view everyday but sometime you know at least at least I see green I see some green and then the sun or rain anything anything outside .. not only in the hospital. [It] makes a difference. … Yeah because it’s always being kept in the building. I’m always .. I can’t really move much so that’s the only that you know .. make me feel like you know .. just cheer me up. To see the green tree .. the car and tram I love it. I love to see and I’m thinking about one day I’m gonna go back to get in the tram again. Just the thing to look forward to. And it also make me sort of feel positive sitting watching…” 2656-09  1.2.6. “I suppose in a small way, [the gym] represents my outside world.” 2656-03  1.2.13. “I go in the elevator. A lot. Which I enjoy. [inaudible] because it's a change of scenery … Um particularly the people coming and going. The people coming up in the lift and some going down and some are waiting and um [laughs] they then I always get a cheery smile.” 2656-07  1.2.3. “[I come to the patient lounge] probably once or twice a day. When you get dead spots and you just want to get out of [the bedroom] and you don’t know when they strike. [When I] wanna get out [I think] oh I’ll do a loop of the ward in [my wheelchair]. Give it a run. … Something different. Occupy your head. Because what’s to occupy your head in here except beige? Oh they’ve got games everywhere if you want to play that, but I’m not really into that. I don’t read any books. Might watch a bit of telly. Trying to keep up with current news.” 2656-01  1.2.15. “I think I’m happy with everything in the gym. It’s great. Some of them [the equipment] I haven’t been able to use them but I think it’s quite good. Yeah and I was I’ve been imagining that one day I’m gonna do that. I can do that. … positive feel positive that one day I’m gonna do this and that. … It’s so .. give me ah .. a stronger attitude to work on my body it does help … It’s a big place. It’s a big room and there’s a lot of equipment. I feel oh this is great mm this is what a gym should be and also the equipment is good too. Help exercise” 2656-09  1.2.25 “Q: is there anything in this room that helps you to feel motivated? A: The steady.” 2656-16  1.1.12 “Well it’s a very good gym. It’s excellent. It’s very large ah I suppose spacious would be the best word for it. You don’t feel hemmed in. The staff are very very good and just a pleasant place. Although I hate gyms! [laughs] … it’s so spacious and it has a lot of windows, and you can see out wide wide … To me it’s just spacious, very clean, and lots and lots of windows” 2656-19 | 1.2.18 “Just like it’s something to look out on. Whether I sit this particular way and I can look down the street this way um or just even I’ve noticed there’s been movement in a couple of the rooms across where I am and I’m thinking oh ok so it seems like there must be a ward there of some description, not knowing the hospital, but I think oh yeah well there is movement, there is other people [small laugh] around um yeah you just don’t I don’t feel like lonely yeah yeah … Can see others aren't that far away really, they probably are, but I think on no they're okay. They’re doing alright over there and I’m doing alright over here and yeah yeah … no as I say I’m happy in the room because I can look out at different spots as I say whether it’s across to another ward which I presume it is or down the street this way or yeah there’s just something that I can look at and that just seems to take your mind of whatever else is going on” 2657-03  1.2.19 “I [think] the windows [are] good you could actually see things had a bit of a view … you don’t feel so isolated I spose, feel more a part of things” 2657-04  1.2.20 “downstairs when me wife comes we go down and have a coffee down on the ground floor [I like going down there] it’s nice [pause] doesn’t make you feel like you’re in hospital … you see people come and go and you know yeah sort of sort of feeling normal … you just feel normal, feeling of normalcy … [you could bring that sense of normalcy into the ward] if you could access some of those services from your bed I suppose like if you wanted coffee or whatever you could just go onto that thing [patient entertainment unit] and order a coffee and have it delivered or whatever. You know what I mean? Yeah that’d be good. [But in the café it’s] a bit of everything I think. The people and the trees and the yeah and the environment itself is quite nice … One thing they could have down there is ah perhaps like a barbers shop or something so you could have a shave and you know a bit of trim and whatever, and they could have the same for women. I mean if people feel better about themselves they’re going to get better.” 2657-04  1.2.20 “Yes I sit there [at the window in my room] and have breakfast and I look out and watch the people going to work. Or about 5 o’clock I’ll sit up to see if it’s daylight. They put the blind back I can see the daylight. … I sit up and have a look see if their lights [in the other hospital windows] are on that gives me an idea it’s about 7 o’clock of a morning. Just have a look. … And if I sit at the window I can see people moving around in there. … it’s just company. You know there’s someone there walking around.” 2657-08  1.2.21 “You got plenty of daylight coming in [to the seating area in corridor] you can see out. Sort of fills your mind. Occupies your mind. It doesn’t .. you know whereas I mean laying in there just doesn’t … I become bored. Sick of watching TV. Q: What is it that occupies your mind about this space? A:Oh it just sort all the truckers going past when they’re driving. Yeah and then a helicopter came .. I watched that yesterday that was good. They come down and they take the patient off and take him down the ramp here and then when they came back they flew off like that and then went choo and then straight out there heading .. it’s amazing I’ve never seen a helicopter do that before. … It fills your mind it keeps your mind active.” 2657-12  1.2.26 “Q: So you’re looking forward to having the breakfast group? A: Just something different. Get out of the room and see different people.” 2657-08  1.2.28 “I get motivated whenever I walk in here .. hospital knowing that if I work hard at improving myself I can go home” 2657-14  1.2.9. “I think it's great that they’ve got the ipads … I like doing wordsearches and I haven’t had any interest since having the stroke to do a word search at all. Um and then I saw my grandchild actually do one on the computer, so I made it that was my mission the next day to go down and to see if I could find it or do it whatever, and I did it and yeah” 2657-03 |
| **1.3. Nature and the outdoors are means of escape** | “I’d like to just go out in the fresh air and go for a walk and you can’t do that.” 2656-18  1.3.1. “But if you can go anywhere out of the pris prison great to anywhere any where where you have just air and walk and seeds … Get out of the prison … don’t want to become, what’s the word, an institution! That’s what I mean! You don’t want to be like an institution. You want to be freedom.” 2656-11  1.3.3. “When the sun’s out you can sit over there [in the outdoor café seating area] or you can sit here [in the therapy garden]. We usually just sit here. This is much better than sitting in the room. It is much better than sitting in that dark room. … The fresh air. The green grass. The the seats and everything. The open air. The open air that’s what I like. Freedom. Everybody likes freedom. That’s what I like. … It’s much better here. People can relax and unwind. Filled up with birds. … I wouldn’t change this [therapy garden] because I think this is nice for the people, I think it’s more freedom away from the hospital … And if your family’s there. My son and I were coming here a couple of times and just sat here … And there’s been plenty of room upstairs for the ones that wants to buy coffee see there’s plenty of room because people come and sit in here. It’s nice isn’t it? Nice and quiet yeah. It’s nice. I like it. … See they [other patients] like to come out here. They like to come out of the hospital. Yeah and have a bit of freedom. There’s nothing better than freedom.” 2656-08  1.3.4. “so that’s see that’s we go out of the cafe … and so you can walk around there but it’s all a bit dark … you sit there and you know there’s no real sun” 2656-02  1.3.4. “the other day my daughter comes in it was a beautiful day what was it? Sunday, Sunday it might have been. She said come on Dad we’re going for a walk in the courtyard … So off we went down there and the sun was coming through and it really felt good. It really sort of felt like saying I’m alive, you know? This is still happening outside. You know? It was very satisfying. We were only there for about half an hour, forty minutes sitting there but nah it was yeah it was very good.” 2656-04  1.3.5. “about the room .. it could be more light because when it’s light around around you um around us ah you lighten up ourselves too. If it’s dark it’s dark.” 2656-05  1.3.6. “when you stand here [in the gym looking out window] it is it’s something something .. gives you the idea that you are .. you somewhere with other people or with .. even if you by yourself you can always look at trees [out the window] .. if there are birds out there it’s nice. There are no birds today (laughs) so .. it’s nice to stay in here and just .. I like outdoors. … I was brought up .. I was brought up um back home we had um a lot of space and we um we were outside all the time … I think it’s it does continue and sometimes I miss the outdoors if I can’t go out. If the weather doesn’t allow or sickness or whatever ah I miss the outdoor life. … If you stay in your room it’s depressing.” 2656-05  1.3.7. “And they wanted to take me – there’s a garden out there is there? [looking at therapy garden] Oh that’s were [son] said he’d take me. But it was too hot.” 2656-06  1.3.8. “A bit of greenery instead of the cement but um no. There’s not much I’d I I just like a bit of privacy you know? … Well I I I’d make it a bit more pleasant you know? I’d .. I’d have some flowers or something like that. Take the hedges out and put the flowers” 2656-08  1.3.9. “even if they had it in pots the plants inside you know that’d be ok … I don’t know and they could put them in the rooms if you wanted to … Brighten the place. Nothing much here is there? There’s nothing much to look at.” 2656-08  1.3.11. “well there where they’ve got that green felt part near the café the café the café and um and we just sit out where the table ah the benches are and um and just and I I’m interest anything in that area. For me, it’s like when I find the ah I say with J that I like pull of the ah leaf and then I’ll smell that and you know scrinch it what’s the word? R: crush? M: Oh you crush it. And sm smell it and then and I’m also interest the weeds in that are ok pull out some wo oh of those, just 2 or 3 or something like that and some of the flowers have been picked up for the weeds and stuff like that and I was walking through the grass finding the seeds that get caught in their socks and stuff [like that]. And just sort of anything of interest … Because there are well because the because you can scrunch whatever these [crushes leaf in hand] and smell whatever the the um scrunch what’s that word I used? R: Scrunch? Or crush? And you can sniff it. M: Yeah at least you can R: Oh that smells great! M: Yeah so that it’s just something to do that’s all. I didn’t do that one, just try anyone of these. R: Mmm it’s a good one. Eucalyptus. M: Yeah. It’s better than not seeing it. R: Yeah yeah. Yeah. M: It’s just a way in the breeze and in the sun. Somewhere to sit and talk about whatever. Chit chats. R: Yes. M: Nothing really but ah. [pause] and so [pause] R: it’s a nice place to come M: And the wattle that’s been taken out. That’s what’s the word for it um.. no one planted it it just R: oh it seeded itself M: yes, right there R: self-seeded M: Yes I can try the leaf that one. It won’t smell anything but that one I’ll try. It’s better than not doing something that’s all. Than just sitting in a prison. But that one I think doesn’t smell any one R: It doesn’t? Let’s see. Mmm oh no I don’t think so, I can just smell the eucalyptus on your hands. … Thanks for the sun” 2656-10  1.3.12. “Oh I like I like sitting outside. I like being outside anyway right. Just being outside having a coffee or a cup of tea. Just sitting outside and I’ll feel better than being inside.” 2656-15  1.3.13 “found like if I sit sat kind of on that area [looking out the window in bedroom] I didn’t see the building and the this area over there which I used to say it was like Tuscany because it was um this tall pen pen pencil pines and gardens and and the um that sort of at dusk it was just beautiful and I’d sit there and it was just perfect. And sometimes I would just look at the clouds .. that one [new room] really hasn’t got a great view.” 2656-18  1.3.14. “It’s pretty stark [in the café outdoor seating area]. But that doesn’t really matter. Q: Mmm what do you mean by stark? A: Oh well the tables are very sort of ah they’re grey and it’s not I can’t really think of the right words ah but it’s nice to be able to go outside and sit at the table there. The sun and the breeze Q: mhm and you said that it feels a bit stark but that doesn’t really matter A: Well it’s grey Q: Yeah. But you said that it doesn’t really matter that it feels stark. So why doesn’t it matter A: cos I don’t care Q: Yeah so what’s more important than A: A good cup of coffee and the sun Q: aha yeah A: And the ah being out in the fresh air.” 2656-19 | 1.3.17 “being able to look out [of the window] and and sort of see the environment around ya, and look down and you’ve got gardens and things it makes it more pleasureable. It’s not so barren, and it’s not institutionalised if you know what I mean” 2657-06  1.3.18 “there’s no shade out there at the moment and I wouldn’t go out in this heat” 2657-06  1.3.19 “[in garden out front of hospital] I love the outdoors. I don’t like indoors. Never have done. … [I’m] an outdoors person … It’s lovely out here. I mean, um, can sit in the shade. But you just look and there’s nature. There’s flowers. There’s treesen. So they breath the oxygen and um Yep and and it’s laid out beautifully.” 2657-06  1.3.20 “A few pot plants down the hall might help but then you’ve gotta find a space for them too .. some palms or something I don’t know.”2657-09  1.3.21 “I would enjoy going out. because I’m an outside person (laughs) … I’m just an outside person. I could say inside, I’m happy in my own home or wherever but that’s why I’m happy here because I can see out [the window]. … But if you’re stuck in your room .. I hope I never go to gaol .. I didn’t think I could cope with that (laughs).” 2657-09  1.3.22 “it’s not stuffy. Yeah it’s open. You got plenty of daylight coming in you can see out.” 2657-12  1.3.23 “I like the .. view [in my bedroom] being up higher [i.e. on a high floor of the building] and .. [pause] and it’s generally nice and sunny here. I like lying in the sun.” 2657-14  1.3.24 “The shape of [the hallways] I just can’t stand the shape of them … [pause] don’t know just don’t like them … I dunno I spose cos we’re boxed in if you know what I mean? … At least once or twice a week … If I could walk outside here you know just … Cos you know it it’d be more more handy for us you know just be getting out and around … Wouldn’t wouldn’t make me feel as if I’m enclosed if you know what I mean … I I just can’t I spose it’s claus claustrophobic in one way but it’s too restrictive if you know what I mean … [I don’t do any] physical activity at all … I spose I’m more of a country boy than anything … As I said you got to be able to move around the place outside occasionally. It’s too enclosed.” 2657-15  1.3.25 “the weather’s been pretty dismal so it keeps me inside.” 2657-16  1.3.26 “it’s good to be outside, out of this place. Lifts the spirits a bit. Like I said it’s like a jail up here sometimes so it feels good to get out of here.” 2657-16  1.3.27 “just peaceful being able to look at the mountains [out the window]. Makes you wanna go out there. Gives you a bit of inspiration to wanna go outside. Wanna go and do something.” 2657-16  1.3.15. “I don’t think I’m allowed to yeah [but if I could go anywhere I’d] probably to get outside maybe. Yep, but on the same token I’m too scared in case they said and I’m too scared to go on my own anyway, but um yeah that might be the next venture that I just go out say for an afternoon. Even if it was just to sit on the grass. If there’s grass there, I don’t know if coming in here I saw or grass or not. Um yeah. So that’s probably my next aim yeah is just to get out. Mmm Probably get my bearings more so than anything yeah yeah” 2657-03  1.3.16 “[If I could change anything about my bedroom I’d put in] A few skylights a bit of natural light instead of what they currently got … it’s all artificial light … And the only natural light comes through there [window] and that doesn’t go very far.” 2657-04 |

**Theme 2 - Power, dependency, and identity in an institutional environment**

| **Sub-theme** | **Case 1 quotes** | **Case 2 quotes** |
| --- | --- | --- |
| **2.1. Surveillance** | 2.1.1. “You’re in the colosseum and that’s where you are. … You learn that as you go along, you gotta change your thinking when you’re in here.” (2656-01)  2.1.2 “I feel cooped up. When you’re told you can’t do something and you’re 72! … I’ve always been a very independent person. I do what I want to do when I want to.” (2656-12)  2.1.3 “they don’t want me to leave the hospital because they feel it’s too dangerous for these people in charge and um so I think I’m like forever stuck in this hell hole. … They think I’m gonna .. I’m falling all over and I’m not ah and I haven’t been blessed with a .. you may go, you may leave now [permission] … It’s like they always think I’m gonna make them um get get news report that says ah … they made me fall or I made myself fall” 2656-20  2.1.9 “[I] sneak out. Don’t you tell anyone … [I] just sit down there in the sun … I actually walked across the road the other day. Traffic’s holding off [inaudible] and then a truck came bolting up but luckily it stopped and as I was walking across the road[inaudible] it’s very obvious I’m a patient dressed like this. … [I go outside to] just get out of all this you know there’s too much watching. It’s like being in school … I can’t get out of here without she’s got to sign me out and sign me back in. No that’s the doctor being [inaudible] … [sneaking outside is] a release. No body around to tell you what to do. You can’t go far unless you’re taken. And to make sure I can’t go far [name]’s got my wallet. But I’ve got a legacy taxi card. So all I’ve got to do is ring the taxi and they’ll come and pick me up and get to the other end and then I pay him.” 2656-12  2.1.29 “I don’t think anybody [i.e., any patient] in here wants power. They want respect. There’s a big difference.” 2656-01 | 2.1.5 “It’s very depressing [in the hospital]. I just find it depressing. I find it makes my mood depressed. … It feels a bit like a jail. Just the atmosphere and that. And the colour scheme. It needs a bit more colour. Make it a bit more lively. I don’t know red, blue, green. I don’t know .. the staff rushing around doing their thing [makes the atmosphere feel like jail]. Patients just walking round with their heads down. Not very cheery. Everyone seems to be down in the dumps a lot. That’s fair enough I suppose nothing to feel happy about in here. I get where they’re coming from.” 2657-16  2.1.6 “it’s like a jail up here sometimes so it feels good to get out of here. All the rules they give me that I gotta live by. I feel a bit singled out sometimes so I don’t know you giving anybody else these rules or is it just me. … It makes me angry and depressed. Cos I hate being locked up in hospital and I don’t see why I should have to be locked up. I’ve done nothing wrong. I haven’t committed any crimes. I just had a stroke which was out of my control. It just happened to me. And I get locked in here like I’ve done something wrong.” 2657-16  2.1.7 “I really gotta behave myself cos I didn’t behave myself there very well so they said if I don’t behave myself they won’t [discharge me]. So now I gotta pull my head in and really behave myself. Otherwise I won’t get home which is a real bastard. It should go on your health and stuff .. if you’re not healthy enough to go home, but I wanna go home.” 2657-16  2.1.8 “I tell you what, it’s like jail … That’s exactly what it’s like. I’ve never been locked up before in me life and I don’t intend in being locked up but I reckon it is exactly like jail would be like … Everyone’s keeping their eye on everyone no matter what. … What’s wrong with me going [outside the ward] by meself though? That’s what gives me the shits. It gives me the shits … I shouldn’t have to ask! That’s what gives me the shits.” 2657-15  2.1.27 “they have security … That makes me feel a little bit safe knowing there’s security there … I talk to them quite a bit when they’re around. I get along with them well. They good blokes. … he used to hang out outside my room a little bit. … I made some comments about the nurses and the doctors so they made security stand outside my door or sit outside my doors to make sure I wasn’t going to hurt any body. Yeah they had three of them there for a little bit. I don’t know why they need three security guards for a bloke with one arm and one leg. But anyway they can do what they wanna do. They were earning money for doing nothing I wasn’t causing any trouble they were just sitting in chairs. They probably made good money for sitting down all day so there you go. And we all got along well. Have a little talk and a laugh every day. … I knew nothing was going to happen to me while they were outside my door”2657-16 |
| **2.2. Relying on the environment for safety and well-being** | 2.2.7 “See the colour of those doors? Beige. The whole place is beige. … It it just flattens you. … Beige. It’s just that’s all it is … There’s nothing out there to amuse you it’s just all beige.” 2656-01  2.1.15 “that’s why I asked if I can walk up and down the stairs [because the lifts break down]. But no I’m not allowed to do that. Yeah. … I just thought it’s only, like to go down to the, it’s one floor down you know, two floors down sort of thing. And they said no! And I sort of understand because a couple of the ah a couple of the stairs are quite st st s steep. And I said look I I’ve gone through a lot of that but it you know when I I went with H [name of OT] once, I walked down and walked up, and I she said and I I I said look I hold on to the the [handrail]. … I asked if I could go to the stairs, “look we’re sorry, you can’t” you know “but this is the reason” and I go oh that’s fine thank you. And you know I think they’re all surprised that all I need to know is why.” 2656-02  2.2.8 “got no pictures [in the bedroom] or … they could have put something something light up the place but who knows why they can’t do it. … Um a nice picture like the one outside [in the hallway]. Other pictures probably .. even um children’s um paintings” 2656-05  2.2.41 “It would be good if you could walk around but you can’t because they’re all too busy.” 2656-08  2.2.38 “[I want] decent television [in my bedroom]. With something on. I don’t wanna watch cricket. … Television in .. they gotta better picture than we’ve got. [referring to TV on wall by chairs on ground floor]. On our TV we haven’t got any choice only cricket. Cricket .. got no videos .. nothing .. cricket. Old people don’t like cricket.” 2656-08  2.2.47 “It’s only like one time we had experience with the .. cos I lost the buzzer and then there wasn’t anyone coming the whole night and ah .. and I need to go for a pan or get a bed pan or something there was no one come and then eventually and take like the whole night and now change they coming round every 15 minutes or I mean a half an hour … that’s the thing that should be if you don’t hear anything from the patient I think you should you know keep your eyes on and go in and check them re.. whether anything went wrong or anything right that you can look on now so it can’t be right the whole night there’s not going to call anyone or the whole day is no one come. It’s not right. … sometime you might need something that you just can’t reach these things and it happened because it’s dropped or yeah and I feel you know regular regularly check up it’s safe it’s safe here and I’m safe.” 2656-09  “Yeah so I loved about having my my for me about having my iPhone. … I’m all [laughs] always all the time on to um I can be on ah I ah internet internet. So that’s one great thing for um. So I can look at internet. And also phoning and ah text and internet. And so and I ideally would be like like deck sorry top desk or whatever the word it. Desk top. That’s be great and then you’d be able to have a a an internet commuter” 2656-11  “I quite like [the gym] it being light and airy and a big space. … it’s better than being gloomy and miserable … At home we’ve got lots of windows. And you know it’s a very light house. It just sort of feels familiar and there’s views out of the windows. At home we’ve got views out of the windows. That’s one of the best things about home. So that’s why it’s a nice feeling and we’ve got a timber floor at home and it’s a timber floor [in the gym]” 2656-16  2.2.9 “There’s a an SBS channel called chill which has music to unwind. You’re meant to .. when you get home from work you turn it on and relax so I often listen to SBS Chill [on my headphones in my bedroom]. … it has a certain beat that makes you feel relaxed. I find it I find you know very soft music soothing.” 2656-16  2.2.48 “Yes well I like to feel as if I have still little bit of control. Q: Mm. Ahm yeah. A: But it’s very limited I know. Q: Well are there other things that help you feel like you have .. you know .. help you utilize the control you do have that you’ve noticed? A: Say that again. Q: Are there other things that help you to use .. make the most of the control that you do have? A: Yes yes the bell I won’t go anywhere without it. Q: Ah the bell yes. Yeah, yeah. A: And to me it’s like at the moment, it’s very important with that bell. Try not to use it but at least it’s there and I don’t feel .. and I’m not saying, alone is not the word I don’t know the word but if it’s there I feel as if I have control. Q: Yeah. Yeah so are you saying alone is not the word? Do you mean because you don’t feel lonely is that right? A: No I don’t feel lonely. I just feel connection.” 2656-17  2.2.49 “Yes [I feel safe]. Because if I don’t I know this [buzzer] is here. … Yes I don’t without that I feel I’m cut off. This is my real friend. … Whether four walls make you feel safe I suppose they do but this is a real thing in my life. That I don’t feel as though I’m alone.” 2656-17  2.2.11 “Just make it look a bit .. not so stark. Paint [the walls] a bit (laughs) m maybe some plants and um some flo flow [flowers]. Yeah and photos. Especially I think in in the the ro [bed]rooms.” 2656-18  “I noticed the other day there was a day in in the ward that there was some music on. And I thought they should do that through the day. Not all the day but … Mm you’d think actually they might always have some music on.” 2656-18 | 2.2.12 “Just add some more colour. Maybe go to the local pre-school and get some paintings from the kids there and put them on the walls. I think the elderly people would like it too. My dad’s seems to like when my nephews and nieces do pictures for him. He puts them straight up on the wall and he’s 80. He likes the colour and just the abstractness of them .. you don’t really know what it is. It’s all over the place but they look good.” 2657-16  2.2.13 “There’s usually pretty good music going on there [in the gym] so you’re motivated or I take my own music. I usually listen to music I like [in my own room so I feel motivated]. … Something to get you a bit more inspired maybe the music they play .. they could play bit more inspirational music [in the gym]” 2657-16  2.2.16 “I didn’t go for a walk because I have to walk with a nurse you know. Limiting yeah.” 2657-02  “[The patient entertainment unit] could be a little bit more um updated like if they had Netflix or something on there if you could watch something like a series or something … [Whether it’s easy to use is] a little bit on and off, hit and miss, needs to be a bit simpler. I don’t know how older people would get on trying to use it you know” 2656-04  2.2.22 “Knowing that the nurses are just out the front if something goes wrong, or just a buzzer away [makes me feel safe].” 2657-14  2.2.30 “I’m very pleased with it but I like the sides of the bed I pull ‘em up cos I’m frightened I’ll fall out. Yeah I pull them up.” 2657-08  2.2.31 [daughter (D) of patient speaking – the patient (A) also agreed with what she was saying] “I mean I think well although they’re clean they’re very sterile. There’s .. like I brought flowers that got thrown away. There’s no .. there’s just very calm colours but I think it would nice to have some art work on the walls. Sort of just a bit of stimulant .. cos you can watch TV but I think sometimes you might just like something like a bright picture or something to look at and just some stimulant in here. A: Well when you first came in the lift when I was downstairs I see pictures D: Yeah in the hallway A: Yeah in the hallway. You see you could do it in the [bed]room. Which would be a help for patients. D: just a bit more stimulant I think. Yeah. … A: Well I’ve only got the thing there to look at [hospital chart on wall]. … D: but I think having a bit of like colour. You know just dunno just a picture or something it it takes away the starkness at times. It can just give you that warmth. It makes you feel a little bit more better. Think like even like an outside picture, if you can’t get to the window, if they had something like with an outside .. flowers or something.” 2657-01  2.2.32 “Oh I think that [the bedroom is] pretty nice because it’s got red and gold. It’s bright isn’t it? It’s got the grey and everything and it’s got everything in it and all the equipment you know is in it. It’s modern and .. don’t know what words to use but it’s just a nice room. … I suppose it’s reassuring in a way .. Only because it’s modern you feel safer in it .. it’s got everything in it that you need. All that you can have in this present time. … [the colours are] bright and cheerful and they brighten up the grey and the brown. They all just sort of blends in nice doesn’t it? … It’s just safe. You know. You don’t have any ill feelings or anything. Yes it’s good in that way.” 2657-02  2.2.33 “I like it because as you say it’s everything still seems all fresh and it’s all new um doesn’t look weather in any way at the moment um [pause] Yeah. … because I think that also can affect your the mood that you might be in. Yeah like well when things are like fresh clean and that you just think oh yeah I feel good in myself, whereas if it’s dull and and doesn’t look overly great you think oh well you just tend to go down that dull kind of track.” 2657-03  2.2.34 “I think the equipment they’ve got and the set ups they’ve got where the rehab and the gym and the pool and all that to know that they’ve got really good stuff, that’s fairly motivating you know. I mean if you walked in and everything was dirty and rotten and falling apart and that that wouldn’t be inspirational or motivating, but everything seems to be pretty well state of the art you know which is good … like they know what they’re doing” 2657-04  2.2.35 “It is a very nice room it’s kept very clean. It’s beautifully kept. I’m amazed at how clean they keep this place you know. … I I’m amazed at how clean they keep it with the amount of people going through. The toilets are kept very well. That’s something I’m very fussy about (laughs). … I’m really amazed when I walk through anywhere, this place is so clean, it is remarkably clean. … [It makes me feel] confidence in the place. You think if they have toilets then they’ll have a clean kitchen. Things like that passes through your mind.” 2657-09  2.2.36 “It’s a very pleasant room. It has a good view outside. … it’s not an interesting room. … It’s a very disinteresting room in itself. It’s got this beautiful view which overshadows everything else. … I find that end room boring with the grey. I’m not a great believer in grey paint … but if you put something like that on it it takes it away … I thought about that one day but I don’t know what I thought now (laughs) um something um well there are blue and greys outside .. maybe a pastel colour .. with you know a a light ceiling to reflect … No [the grey is] good actually. You can be in a hospital and getting a lot of use. You need to account for all those extra feet that come through haven’t you. … I don’t like greys as a rule but in here I can appreciate because they go with anything.” 2657-09  2.2.37 “I like the way it looks. … It’s clean. It’s clean, it’s healthy. You know looks new. All new. Yeah. The colour coordination is good .. grey and white. Yeah no worries. Lighting’s good. The lightings good. Yeah they did a good job of that. They made sure what’s that when it’s night that the lighting’s good … Yeah. It’s pretty good. Colourful.” 2657-12  “[When I’m in my room I] just watch TV and listen to music. Get on my phone, text people. Call people. … I’m trying to get my laptop repaired at the moment. I want to get some memory type games to put on it so I can help um build up my memory again. Work on me memory. But um I forgot what’s my passcode to get into it so I’ve got to pay someone to get someone to get pass the passcode and put a new one in.” 2657-16  2.3.13 “Because of the way the bed is originally the mattress which has been changed which I appreciated very, very, very much don’t forget that bit that’s very important and the pillows are good. And I said the linens are always lovely and everything’s very clean. Um I think it’s probably just me I’m very restless .. being away from home I’m extremely restless and you don’t actually have what .. I can remember as a child being in hospital, the nurses would go past very quietly make sure you were alright and you were settled. You don’t have any of that in the adult places apparently now but it’s reassuring if somebody says are you alright love and you say yes thanks. Especially if you’re not well. When I’m terribly restless I get so tangled up I have to press the thing [nurse buzzer] to get someone to help” 2657-09 |
| **2.3. Ownership of the environment** | 2.3.2 “you go down to the reception area you got plants and cards and that to brighten it. But we got beige.” 2656-01  2.1.22 “you know first they didn’t explain it anything to me, and that’s the other thing for me, if you tell me even if I think it’s silly I’ll just go oh yeah well that’s what you have to do. … they said well you certainly can’t go outside, this is Melbourne, you know, and you live in Canberra. [laughs] And that really… so I said well in actual fact I bet I could tell you more about this area than you can. And so after that they’ve been quite nice to me. And I and if they’d said there’s a a Victorian government rule that ah you’ve got to have somebody with you because if anything happened I would have gone, they would only have had to gone there’s a rule and I would have been. But but I think I don’t know why they were trying to tell me something that that probably was right, but if they’d only just said well this is the rule, I would have gone that’s alright I don’t need anything else.” 2656-02  “I’ve come to realise that having a stroke is more than just losing muscle power; it seems to alter one’s relationship with the world … my relationship with the world I had taken for granted and all of a sudden was no longer there. … In the past I have been able to get out of bed, go anywhere in the house, wherever I want when I want, but I can’t do that now.”2656-03  2.3.17 “I think people will come and go and this is the room, this is the way the hospital they’ve got it set up to their ah to best advantage you know. Patients coming in and out.” 2656-04  2.3.18 “It’s amazing you know how people once you’re in hospital where they put your change of clothes that’s where you classify as your home. It’s you know, funny thing, but that’s how it is. At least for me that’s how I feel and I think a lot of other patients you know yeah home is where you put your slippers under the bed that’s home aw yeah. Ah yeah but ah you know it’s a bit of a sometimes it is a bit of a hard pill to swallow it’s not easy.” 2656-04  2.3.21 “Access to everywhere is pretty good. … People are pretty much welcome to walk wherever they want and if I want to go down to the gym I can” 2656-04  2.1.18 “I wasn’t allowed to go [to the ward lounge] by myself I wasn’t .. I am now (laughs) but I wasn't … I don’t know if um if they’ve got facilities here ah .. to go out of the room and meet other people .. I don’t know if they've got the facilities or if they're allowed to do that … the doctor or could be a nurse told me that I’m allowed to walk around by myself cos he said now you can walk you can go up around here” 2656-05  2.3.15 “Everything’s perfect [in the gym] for what I’ve gotta do.” 2657-08  “I’ve um never taken any notice [of the building]. I never take in any notice. I just wanna get better. I don’t take any notice of things. I don’t even look at them. … So I don’t wanna go anywhere else [in the building]. I wanna go home and when I finish this hospital .. I don’t want to see another one. And I’m sure the hospital and the doctors would understand that because that would mean they’ve done their duty and I’ve done mine my duty. … Just to get out. Get out. What is there to notice? The curtains. The doors. The young man walking up and down. What do you .. what do you have? There’s nothing here. But what can you do? Nothing. Can you?” 2656-08  2.1.28 “also it’ll be OT soon that’s why. I’m sitting waiting for them cos I don’t want people to wait for me.” 2656-09  2.1.19 “[the lounge room] doesn’t look well used though. Maybe cos no one knows it’s here. Probably only used by staff. … the staff are keeping to themselves maybe. But for people like who are not allowed to leave the room, not allowed to walk, they would keep it a secret. They were paranoid about me even putting one foot on the ground cos I might fall.” 2656-10  2.3.25 “come out and give me a lecture and tell me I’m a naughty person like this .. when you’ve got the ah night extra large bag on for the urine and you get your foot tangled up in the in the long plastic tube and you buzz them .. he’ll come out and say um now you’ve got to remember this is mine not yours … we’re the ones who get to play with the bags .. we’ll come and empty them they need emptying, it’s not yours to play with. You keep playing with it and get tangled up in it” 2656-16  2.1.34 “I don’t know I probably can just go for a walk but I don’t know and nobody sort of said that you were allowed to go out.” 2656-18  2.3.1 “I don’t care [about going outside] because I’m just passing time in here waiting to get out. Because surely it is going to happen before too long and if it doesn’t then I’m going to despair (laughs) that would be difficult … I do like being out in the sun … [but] I think I will be home within a few weeks. So I’ll leave all those joys for that” 2656-19 | 2.3.19 “I do feel this is my room because I’m paying for it medical-wise” 2657-01  2.3.7 “they swapped them [my bed] over. The nurses on last night was .. one of them put this bed in here for some unknown reason .. the other one [bed] went out. … I don’t know why but the nurse that was on before her later night insisted the other one [bed] come in and she got that ah water bed mattress for me” 2657-10  2.3.8 “I can’t take you down [to the therapy garden] cos it’s um you’ve gotta have someone to unlock the door” 2657-06  2.3.10 “As for the set up [of the bed room] I can’t comment on that because all I do is say will you please do this, will you please get me that so it doesn’t give you a real feel of the room, but from where I am I’m very happy.” 2656-7  2.3.11 “So I just asked C[name of partner] if she’d get the kids to do them and so she sat the kids down and got them to do some pictures. Which is good. I had a couple more on me door but I don’t know where they’re gone .. from R[name of son]. R[name of son] made me a couple of big pictures. [They were on] this side of the door so when the door was open and I used to come down the hallway and I knew which room was mine straightaway. Now I have a bit of trouble. I gotta wait til I get to the number and see the number. … I don’t know someone just took them down. I was told not to put them on the walls cos it can peel the paint off the wall so I put em on the door cos the door is powder coated. The paint’s not gonna go. … Sometimes they fold em up and put them on the red shelves.” 2657-16  2.3.23 “I can’t reach it that side cos I’m left handed. And if it was there I could reach across and I said to the nurse “who shifted it?” .. she said “I did”. … I dunno [why they moved it]. I went to get some tissues and I couldn’t reach over but never mind. It could be worse things.” 2657-08  2.3.22 “not every picture suits a person. So that’s why I think that [having a space for patients to put their own pictures] is good because you can put up something like that of your family. Or your dog (laughs) or something .. so you can just have a little thing slip up there where you can put a little envelope type thing. And that’s a kind of vision where you want to go to. You know what I mean? … Whereas a picture of a rose or something might be pretty but a rose is a rose as they say. … You can put a picture of a horse up maybe a woman wouldn’t like it, if you put a picture of a pussy cat up there maybe a man wouldn’t like it so .. what can you do?” 2657-09  2.3.29 “it feels a little bit more boring on the weekend. Cos I’m used to doing something at the weekend. Catching up with me mates, having a beer, going for a walk and doing something. Now there’s nothing to do on the weekend here. There’s no activities or nothing. I saw on me bloody poster thing over there .. the calendar said weekend activities and programs but I have not been told about one. No one comes around and tells me anything. There’s one bloke’s come around one time and offered me to play bingo. I didn’t really want to play bingo so I said no thanks. … I want something a bit more sporty, more .. a bit more fun than bingo. Only old ladies and old men play bingo. There is a lot of old ladies and old men in here so I understand why they probably play bingo. … [they need] some activities for the youth. The younger generation of people.” 2657-16 |

**Theme 3 - The rehabilitation facility is a shared space**

| **Sub-theme** | **Case 1 quotes** | **Case 2 quotes** |
| --- | --- | --- |
| **3.1. Compromise, privacy, and peer support in the bedrooms** | “hear that door? It starts at 5 o’clock in the morning. The staff door. It just wakes you up. I told ‘em about it but they can’t do anything – it’s a structural thing. They put signs on roads so trucks don’t use engine brakes yet you’ve got a bloody door like that right next to the bedroom common… It’s just dumb … You can’t [sleep]! You’re waiting for the next person to bang it because it could be loud or it could be reasonable and you don’t know which one you’re going to get so you’re atuned to it and I’ve been here long enough to be tuned to the bloody thing.” 2656-01 – single-bed room, ensuite  “a lot of people come to these two [bathrooms opposite my bedroom] because apparently they’re cleaner than some of the others … I save up until I go to the gym or the um down to the occupational kitchen [laughs]. … sometimes it’s almost like they’re lined up to get into the shower. … during the day they often congregate here … just cos the toilets. But I’ve never said oh will you hurry on or anything like that. But then at night time I can [hear] people I’m not sure in which room and they’re having these deep and meaningful discussions and I think where are you during the day?! But again I wouldn’t go and knock on their door or anything like that I reckon. But it’s only in later in the evening, you know sort of 7 or 8, and their room’s over there and I can hear so I just shut the door. R: So when you shut the door you can’t hear them? A: Oh yes you can.” 2656-02 – single-bed room, shared bathroom  “I am puzzled by the sounds I can hear of other patients … there’s one particular man, I become accustomed to him … he has a lot to say … he’s quite familiar now … yes, it is okay … I have trouble getting to sleep sometimes, I am not so sure why that is, perhaps distraction … I think mostly things around me … sounds that come through the door” 2656-03 – single-bed room, ensuite  “Q: I want to ask you as well about what it’s like being in here in a shared room with other patients? A: oh don’t take much notice really … You make do. You’re in here with two other people. … You don’t worry about it. They do their thing and you do your thing. [Name of roommate] and me we sort of become a bit more friendly. … he says in the morning ‘good morning your majesty’ I say ‘ah yes good morning your highness’ and we have a bit of a joke, bit of a laugh. Yeah and as it turned out he lives in [name of suburb] yeah and I said oh yeah how we go! They’ve found all of us and put all of us together! Yeah he lives in [suburb] and Hungarian by birth. From Hungary. We both come from Europe, somewhere in Europe, you’re from Hungary I’m from Italy … you have a bit of a chit chat and stuff like that. But ah nothing in ah in depth you know” 2656-04 – three-bed room  “my room .. I’m happy. I’m very happy with the four ladies. We can talk during the night .. we can laugh (laughs) we don’t cry. … we always try to find ways to to come close … I don’t know if they allow hah .. if the other people allow us to ah get into into conversation … I always always keep them tight … get into conversation. … I’m happy with them. … I prefer to be with other people. Um it could be a bit better if there were just two [instead of four in the room] but … I don’t want to be by myself. … with two it’s always less talky. … Less noise yeah. … I’m not talking too much (laughs). So of course but I've got somebody there who talks a lot …Q: I notice that you hold your um tv [speaker] to your ear, the the tv sound to your ear. A: Yeah. We got ah ah I’ve got it lowered. I don’t want to disturb the other people too much … Q: Yep. Yep. Have you put any of your your own things around your bed? No. Why not? A:.. not enough room. Not enough room to put stuff up. And ah .. and we have to respect other people, is another thing, because if I put something on and doesn’t suit the other person I don’t want to disturb her. … otherwise it’s enough room. Plenty big room um it’s got those curtains to help people .. lying down .. or .. of .. Q: a bit of privacy? A: Yeah.” 2656-05 – four-bed room  “I do get on well with people in general. … I didn't think I'd like the company. But um well actually I was offered a private room yesterday … but I said I think I’d just look a snob now, I couldn't leave these people … And they enjoy the company. … And the lady next to me there [name of roommate 1] I mean ah we know of people. She comes from up north and so do I. Where we grew up as kids you know. And we ah a lot of the same background as country people. … well and [roommate 2] put her hand on me at one stage and said you know, she said I’ve got a stash of pills at home when it gets too much. And I did, I thought, I’ve gotta share that. So I just whispered to one of the staff.” 2656-06 – four-bed room  “I don't always want to get away from [the sounds outside my bedroom]. … because they let me know someone's around. (laughs) … And there are happy sounds, there are people talking and laughing” 2656-07 – single-bed room, ensuite  “Yeah I feel safe. There’s nothing here .. nothing frightens me in the hospital. … I don’t know whether [it’s the room on] this side [or] this side or what - the swearing! … I don’t look in there so I don’t know who’s in there or anything but just before you come they were swearing bad. Yes so you don’t you don’t expect that … I’ve been in hospitals where young people have really screamed and gone off and it didn’t worry me. I just shut the door … During the night? No um I think everybody’s worn themselves out (laughs). With all the screaming and yelling I think they wear themselves down and .. and then everything goes quiet. It never bothers me you know. And by and by the time I watch whatever I’m watching a movie or whatever everybody’s quiet down. Yeah no it doesn’t bother me because I’m not I’m not sleeping so, it’s not bothering me see. No they’re alright. Mm.” 2656-08 – single-bed room, ensuite  “this room is quite privacy … sometime I’m listening to Buddhist chanting and or philosophy or just meditation or chanting keep chanting on on my own. Sometimes out loud, sometime in my head. … Yeah so I feel privacy this room’s nice.” 2656-09 – single-bed room, ensuite  “The ah the room is is very nice but um the hallway is very noisy at night so I try and keep the door closed. … there always seems to be one who constantly requires the nurse, and always talking and um and always yelling out nurse, nurse, nurse, and that wakes me up every time. … And the hallway out there with the doors being opened and closed it’s very annoying. … must be a door here that keeps opening and closing … The fire safety door must be … makes a distinctive noise. Kind of a squeaking” 2656-10 – single-bed room, ensuite  “the thing I find amazing is um we still haven’t moved away from those pull around hospital drapes. … that’s common to all hospitals and we don’t seem to have moved past that. I I find that amazing that we haven’t come up with a better system somewhere along the line to for privacy. You know like you get situations where if you’re sharing a room with four people there’s going to be disruptions I understand that but those disruptions can be either non impacting or impacting and if they minimize the impact that’s obvious to me in that situation it’s probably better for patients if it doesn’t impact them as much. Um to me it aids their recovery. It doesn’t help the sleep at night particularly if you’ve got you know doors, lights going on and off … an ideal situation would be to be in my own room, yes, that would be ideal however I’m practical enough to realize that I’m in the public health system and you need to have beds available … an ideal situation would be to have one room per patient … my medical needs are low and I’m fairly mobile. I don’t .. to me I’d be a waste of space in a in one room quite frankly. Um ah I don’t think that that’s fair on people that need you know, there’s priorities I understand that too but in an ideal situation it would one person one room but you know I just think that maybe two people per room that size, where I am now, I think two people. … interaction with other patients I believe is good. I’ve enjoyed like [name of roommate 1] the guy directly not opposite me but next door to me but across the thing, an older elderly Italian um nice gentleman. We got along well um and um um I enjoy his company. We talk about things you know um [name of roommate 2] just across directly opposite me ah is about my age um he’s, him and I interact well. [Name of roommate 3] who’s on the corner diagonally opposite is um ah suffering the consequences of a stroke, a fairly severe one so you know and he’s a little bit different. Ah but that’s you know, that’s just the way it is. I think interaction with other patients is good but what I’m saying is if there was two you can still interact. … you know you could still bounce things off each other and things like that and if there was um common meeting area where patients go and happen to have a coffee or whatever” 2656-13 – four-bed room  “I’m happy enough in this room. It doesn’t worry me with people in the room. It does other people but not me. I’m happy in this, yeah cos I mean you could talk to talk to the other folks” 2656-15 – four-bed room  “Um two [patients in a room] might wear you out because I find that even just having a conversation tiring. You know if you were trying to keep up a conversation with someone else all day long you’d be zonked at the end of it all. … you’d hope they were an interesting person. … could be um all a bit wearing. … I quite like [being in a room by myself]. Bit more privacy so I like it better than being in a shared [room].” 2656-16 – single-bed room, ensuite  “there are good things about [the ADL bedroom that I’m in now] but there are other areas that make me cranky. I feel very isolated but I guess part of that is about, you know getting ready to [be] going home … Q: Do you mean at home you would be alone? A: Yeah except my cats … … Q: In the room you were in before did that also feel isolated? A: No I loved it. (Laughs) I mean it was a single room with its um own bathroom and lovely view and that sort of thing. So I could .. I felt more relaxed. I’d sit and just look out or watch the building [construction work out the window] or that sort of thing and I think just sort of felt like if the door was open you know and people were chatting more. Q: Pass by? A: Yeah yeah generally I did like being in there and it, I am just that sort of person that I do like to be very private … I liked my [old] room (laughs). I think it it was just it was comfortable and I didn’t have to think about other other people really” 2656-18 – single-bed room, shared bathroom (moved from a different single-bed room, ensuite)  “I hate having a shared shared bathroom. That’s just me I’ve never in my life shared a bathroom and a couple of times I’ve had, like in the middle of the night, there’s someone who’s just been messed all over the floor and all there and I’m standing there thinking why am I looking at this?” 2656-18 – single-bed room, shared bathroom (moved from a different single-bed room, ensuite)  “from a personal point of view, um which really doesn’t bug me that much, I don’t like having a a shared toilet … I always have to think is there someone else in there before I go. Like sometimes when I want to go it’s very urgent. Ah and then sometimes there might be somebody there. … Whenever I’ve had a single ward anywhere [else] I’ve always had my own toilet. … Q: Would you like opportunities to be able to talk to other people more? A: No not particularly” 2656-19 – single-bed room, shared bathroom | “I hear the door always swinging out shut because as they going in and out all day long and then at nighttime all night. … They go in there, like take sheets and the towels and things like out so that door swings.” 2657-01 – single-bed room, ensuite  “she’s been a good neighbour friend. … The bathroom’s good, and so close. And you haven’t got competition for the loo. … I don’t mind [sharing a room]. Yeah with people like her is good. Yeah her family have been nice and chatted to me and everything.” 2657-02 – two-bed room  “the bell (laughs). Must drive the nurses nuts.” 2657-02 – two-bed room  “I think the like the rooms are good. Um we still have our privacy with the curtains. … I find it hard to have a conversation actually in the room. … normally [my roommate’s] TV’s on very loud. I can’t hear what people are saying. Um in turn I guess I get a little but frustrated with that. Um can’t hear if the phone’s is on vibrate I can’t hear if that’s going and basically I can’t even hear my own TV. … But I do like it from what I've seen, only having 2 or 1 in each room. I think it’s a fantastic idea. Not like having 4 in a room. … since I've been able to walk better I’ve gone and I head to this area here [lounge nook in hallway] – might only be 10 minutes but just to get a breather out of the room – I head down to that bottom room [lounge room] and just have a look at the newspaper or just watch someone walk by … I normally just pop up there to read the newspaper, or or if normally if my family come that’s we head up there because it just seems easier with the children. … I think it’s the patient [in the bed] next door that just affects me! … it’s just frustration because I think, ‘come on like I’m not making a sound’ you know, ‘give me a break just for a minute’ and yeah. But actually, it was yesterday I spoke to the nurse about it. I just said um oh come on like this is just not called for really, and I said and I shouldn’t be speaking out but I said I’m going to because I don’t know how long that I’m going to be in here for, and I don’t want this to be escalating any higher than what it is, so can you please, can she please turn her TV down? Like I didn’t think that was a huge thing to ask, anyway, they turned it down, and it’s been gone ever since. … she’s got problems whatever they may be, I don’t know, um so yeah [pause] yeah I just sort of didn’t want to step out and say oh rar rar rar rar [complaining] but I think oh come on like fair’s fair and it seemed like yesterday every time I went to have a lie down like she would bang her cup on the bench! Oh no please. And it’s like she knew that I was having a rest, because I pull the curtains all the time, and it was like every 2 minutes ‘bang’ on the desk. ‘Oh man oh man’ I’m thinking, ‘no she doesn’t want me to sleep’. So I just got up and thought oh well, I’m not going to have a fight fight over it or anything you know I’ll just get up and yeah come [to the lounge nook in hallway]. … she does look at me a lot. Um you know how you sense someone looking at you and every time I looked over she was looking I just think when I close the curtains well, she can look as much as she likes because she can’t see me [laughs] … you can’t have a conversation with her because she’s deaf, and I just think oh well, yep, we don’t need to look at each other because we’re not having a conversation. We haven’t, haven’t had one since I’ve been in there. Um yeah and then on the same token I don’t want her to see that I’m, that I have a cranky look on my face because she’s banging that damn cup or whatever it may be [laughs] And I just think oh pull the curtains and she doesn’t have to look at me and I don’t have to look at her! Not that it worries me to look at her, but I just oh well it’s just easier.” 2657-03 – two-bed room  “there’s nothing wrong with the room except there’s four of us in here and it gets very busy, especially at night .. buzzers going off everywhere and so yeah gets a bit hectic … you know what’s happening to each person you know basically yeah so it gets a bit personal. I mean their visitors are your visitors in the end. That’s not a problem but there’s bugger all privacy to a certain degree you know. You got these [curtains] here, sometimes people are emotional or they’re in pain or whatever, I mean that comes straight through … you don’t get a lot of sleep, disrupts your sleep … you know, one thing you need in here is rest” 2657-04 – four-bed room  “I don't know what I've done. The last two patients that have been with me [have had] … Alzheimer’s disease … I just ignore them … It’s not my job to look after them … sometimes he’s um getting up at two or three o’clock in the morning … and he often puts the light on when he gets up.” 2657-05 – two-bed room  “that bloody bell [laughs] sorry… Pain in the neck! [laughs] … I’ve got hearing aids which make it worse. So it sounds as if it’s right in my ear … When you press the buzzer it’s just one beep. But the longer the buzzer’s on, the more persistent it gets. … And if anyone pulls the plug it beeps like that too because they’ve pulled it out the socket and that’s ah ah you know if you’re in trouble” 2657-06 – two-bed room  “Last night we had a, we didn’t sleep too well because [my roommate] was very restless all night, but I don’t mind that because she’s got dementia, and I nursed my husband with dementia so I know she can’t help sort of singing out or trying to get out of bed, when she shouldn’t, um so I don’t mind, you know, hitting the buzzer [laughs] to sort of let the girls know she’s she’s on her way out [laughs] … if somebody needs help, you you help them. If you can do it, do it! … and the same if anybody speaks to me, I always speak to them … You do exactly the same when you’re walking around, you say hello to everybody!” 2657-06  “[I feel like I have] too much [privacy] sometimes … [I’d like] someone to talk to, but there’s no one to come. Me daughter’s gone home. … [my roommate] doesn’t talk and when she does I can’t understand if it’s .. she sleeps all the time. She told me to turn me telly off the other night cos I was watching the football, and she said I can still hear it. I said you can’t it’s turned off … and I said to the nurse did she check her telly and mine. She said no they’re both off. But I don’t like to put it on her to annoy her, but then I like to watch the news and things myself so what do I do?” 2657-08 – two-bed room  “I’m quite comfortable in here. Very noisy hallway. It’s a very, very noisy area to be in. … when I was there I was near the nurses’ station and it drove me mad with people all the time but here it’s very, very noisy up and down this corridor. I can but then I go to sleep but sometimes you think oh I wish they’d be quiet (laughs). They’d be crashing things and noisy. I think every hospital should have a little thought about how they can soundproof some of the areas that are public more for their patients. … Or make some of the staff more aware. Particularly at night. … it’s a bit confronting especially when it’s near your door or your wall. [I think] Who’s, what are they gonna do? Are they gonna break into my room or is somebody going to accidentally come in here? … You know you hear somebody out there and they drop something it’s really noisy and you think oh was it? Is there something wrong? … That the volume of the door slamming should be reduced. It should be made clear to people that others are sleeping. Little old ladies not just me. … It’s terrible. It’s deliberate in that they just slam the door and walk down here and another door slams and what do they have to do that for?” 2657-09 – single-bed room  “the first time I come in I was [sharing a room] with an old chap down there. … I got kicked out of there because I I was talking to me wife and me daughter in me sleep … I think I might have been some noise. They shifted me out the next day. [In a private room you’re] sort of your own boss … if you share it with someone else and they’ve got visitors coming in you know you feel a bit out of it. … Oh yeah you don’t want for much in this hospital. … over in those wards [in the old hospital] you’re all sort of being together because four or six beds. I forget how many it was. I think it was four but there seemed to be a lot more people around. An invasion you know but now there’s a lot of private rooms.” 2657-10 – single-bed room  “[my bedroom is] alright [but] not when you’re stuck here on your own. You got other people coming in and then you can talk to people … I don’t like being on me own it’s horrible … That gets a bit boring. … just listening to everything go on [in the other beds] … that way you know you’re not alone” 2657-11 – four-bed room  2.3.20 “I just listen to me wireless [when I sit out in the corridor]. And don’t interfere with the other person in the bedroom. Because I’m a bit deaf I can turn it up a little bit” 2657-12 – two-bed room  “it’s been stuffy [in my bedroom] … cos some of the [nurses] like to have a chat at night so they you know close the doors cos they want you to sleep, you know. They’re not there to chat, they’re there to work, you know, and it makes it hard because you wake up and you’re hot. And you wake up you’re cold and you wake up you’re hot and … it’s very noisy here cos there’s no carpet. … just a hard hard floor and everything bounces off the walls into the bedrooms” 2657-12 – two-bed room  “There’s been three of four changes [of roommates] since I first got here. Not too bad you get to meet different people.” 2657-14 – two-bed room  “Too many fellas in one bloody compartment any how … I was expecting to be in a little you know like have in hospitals a little [private] room like that … [My roommates are] alright there for the first two days but afterwards there’s just … they’d be talking to someone else and you just be hearing them say the same bloody time and time and time and time again … been a pain in the arse” 2657-15 – four-bed room  “If those bloody bell don’t shut up I’m gonna kill the bastard [patient alert bell is dinging in the background] … That’s one one thing keeps a fella awake all night … You should try to sleep with the bastard” 2657-15 – four-bed room  “Oh [my sleep has been] terrible. Too cold and the noise at nighttime. There’s always trolleys going past and the beepers going off. Buzzer things. You can hear it outside the door. Someone’s always pressed it. If it’s not me it’s somebody else.” 2657-16 – single-bed room, ensuite |
| **3.2. Social connection in hallways and therapy spaces** | “they [the other patients] start waving to you through the door, you get to know them … All I see [in the hallways] is a lot of depressed people. One after another. And you can see some in a lot of anguish and some not. And you just go past it to the next one. And then all of a sudden it just goes into a blur in your head and you don’t worry about it. You can’t! You can’t take it in. There’s always somebody worse off than you, yeah.” 2656-01  “I’m chatting to some of them now. Yeah so there’s one lady and a couple of others that I’m doing the gym with and some of that. … I felt a bit guilty cos she’s been here for a while. And so I said look some of them I just can’t talk to. At the moment I can’t even make the, which I would in the past I would have gone around and I would have made small chat, but I can even make that. … the main reason I’m closing [my bedroom] door here, there’s a young man who’s gotta um he’s got an intellectual disability … I know a few people here have treated him fairly poorly … I feel sorry for all of that, but I am just I am not up … I just couldn’t couldn’t cope with somebody just doing doing that. … And that’s why I go to bed [laughs] I go to bed and watch telly cos I just figure cos some of the others have the noise on really loud don’t they and so I just do that, so I normally go to sleep, and then I’ll get up um early and have my shower and then sit and read or sit and do that [points to crossword]. Cos that way I feel like I’ve got the space to myself.” 2656-02    “when I look at some of them [who] I go to the gym and all that with I’m probably physically I’m still probably the most mobile. … [And] the OT kitchen around the corner … it was quite nice and it was actually another way to meet um some there are a couple of quite nice older women that I’ve seen since then that I’ve been chatting to … they’re people who are a little bit more um you’d say physically well not physically able um I’d say intellectually … so that’s a good way for people to talk cos there were one man who’s been who was at a lunch I did … and his wife died you know and they’ve been married for 30 something years … and he’s moved up to Mornington now and he was really happy to talk to everyone about it.” 2656-02  “I remember the last couple of days [at the other hospital] especially the last day we walked right around the hallway of the hospital and there were people everywhere talking and you sort of you know you feel more like alive, whereas here you feel more down. … See now there’s always that bloke [other patient] sitting outside here here, the nurses have had to prop him out there and then wheel him back in [to his room] at night time. … oh the poor fella you gotta feel sorry for the poor bloke. But what can you do? Nothing much. … I’ll walk past and have a bit of a laugh with him and I say I’ll come back out and I’ve got pictures I’m with ya. And he’s happy. It doesn’t take much to make him happy really. A lil bit of a human kindness you might say. Costs you nothing.” 2656-04  “I thought I would be self-conscious of others working in [the gym], but you’re busy yourself and you don’t see them [laughs]” 2656-06  “Oh yes this [chairs in waiting area in hallway] is where we came in, this is where we brought [roommate 2] last night oh yes. She knew this place. It was a nice place to sit. We didn't know whether she would talk or not. And the nurse was so lovely, she came and wheeled [roommate 1] around.” 2656-06  “I was really quite surprised at how much the gym is used. … [I like] the people you meet down there … People like myself. Having troubles and coping with them. … you’ve got some sort of companionship.” 2656-07  “[In the gym I feel] relaxed but motivated. Cos there’s other people doing things at various stages of rehabilitation and ah … Oh just I look out the corner of my eye and see someone better or worse than me and the better ones I know that they have been worse and they’re getting better so that gives me my motivation. But I, my entire stay here I haven’t spoken to anyone else who’s had a stroke cos you’d get automatically compare notes. Oh you’re doing, well better than me or or I’m doing better than you or whatever. It might create jealousy. … I don’t know whether it’s because I’m a loner or not but I prefer to do it myself and not have to share it with anyone else.” 2656-10  “if there aren’t other rooms anywhere you can at least sit here [on a chair in the hallway]. … Just a different place. … and you can see the food sta staff … I can see them coming and going coming and going coming and going” 2656-11  “all the different sort of people [in the gym] all the time some of them can hardly walk so don’t do much … Some of the poor buggers will never have much future … [makes me feel] pretty lucky I s’pose” 2656-14  “Here [in the gym] there’s you know quite a bit of activity going on generally usually a number of people doing things. And you see a number of people who are in similar situations to yourself and now they’re walking and now they’re doing things so that’s very encouraging. You know that it does work. Like in the occupational therapy class there’s another guy who is using a knife and fork to cut up plasticine. I said how long have you been doing that for? He said it’s taken me a month to learn how to do this. I thought oh well there you go if you stick at it you can do it. So it clearly works for some people. There’s people in there that you see that are learning how to walk. They’re going through the same things that you are going through and they’re doing it so you know it can be done so that’s that’s very positive reinforcement when you see people down there doing things or doing no better than you are. So you know everyone’s in it, and they can do it. It obviously works if you keep at it.” 2656-16  “every time they take me to the OT room I think wow. One because of the lass [name of OT] and her approach and warmth … But I don’t know there’s something about that room whether it’s the personality you know the person in it that makes the place doesn’t it?” 2656-17  “Well [the gym is] not ever crowded. And everyone’s doing their own thing with their own ah attendant putting them through their paces. I don’t take much notice of them” 2656-19  “The gym? Well it’s sort of too big and that for me to ah join anything” 2656-20 | “If I recall a particular nurse if she might be on. Not to go and see her, but I think oh it’s nice just to say hello [when I walk in the hallways] … Just to say what their feedback to you might be. ‘oh it’s great to see that you’re walking better than yesterday.’ But then one of the nurses this morning said ‘oh you're very unsteady on your feet.’ I'm thinking um I thought I was going really well! [laughs] But yeah um just their feedback as well just to see what their reactions might be in regards to your progress and how you’re getting along. … I try and make it each morning first walk just to the little table that’s here [in the hallway], cos I think it’s quite pleasant looking out [the table is by a window], um and we come at night, I come at night time to this area [lounge nook] with my younger daughter, um probably 20 minutes, and um yeah it’s sort of enough for her to have a bit of a look around as well as I have a look as well.” 2657-03  “love it in [the community room where I have breakfast group] because everybody’s friendly. You talk to everyone. Um and you see how they develop … And you make friends. You you do. You really make friends. Um I’m one of the lucky stroke victims. I wasn’t as bad as some of them. But you see the progress of the ones that that um you see the ones that um really do improve. Like seen one today that’s been coming to physio, and he could hardly walk 3 days ago, now he’s walking around with a walking stick” 2657-06  “my next door neighbour [in room next door] while he was here J[name] he’s gone now, he’s gone home. … He’s not here any more but I got along well with him. … he’d normally pop his head in the door and say goodnight and good morning. And then I would reciprocate it .. good morning good night. … And when the kids were here he would pop his head in and say g’day to them. Yeah he was pretty good. A good bloke.” 2657-16    “It’s just a gym … when I was in there by myself it’s good but it’s when ah I don’t know … too many people” 2657-15  “I just realized how one young man worse off, one of the worse off people than what you are, I went shit when we went to the group therapy I went Jesus Christ poor bugger. I was sitting down and he was sitting over here in that chair and I just walked up the stairs and done that walk down the stairs um .. and worked on the punching bag there … Lot worse than me you know. … If I can get down there than I’m in a better state than what they are. It’s not good but … They’ve got some guts. … You know and then when you see the, the people see you they’re all giving you you know praise, not praise they’re giving you motivation. Saying have a look at you, geez you know cos they seen you when you’re so sick. … it’s a motivation to them too. … Yeah and they’re making you feel good. You know they’re making you wanna get up off your arse and do things and not lay in the bed you know. It’s easy .. it’s easy when there’s a light at the tunnel” 2657-12  “One of the blokes W[name of security guard] I really got along with really well. He ah got hit by a car or a truck when he was on his motorbike and it took out all one side of his body with burns so we were in the same sort of similar situation, he couldn’t move one side of his body for a long time. And I can’t move one side of my body so we had that in common. So we used to talk quite a bit and he was a good bloke. Got on with him well. They were all pretty good blokes but it was W[name of security guard] that I was getting on with really well.” 2657-16 |
| **3.3. Seeking defined communal spaces** | “When [my wife is] here we sometimes go here or go downstairs to the canteen. … [and] I might just sit here [in the sunroom lounge] to have a talk with a bloke …” 2656-01  “my sister walks me round and we went down to the um sunroom which is you know quite pleasant but you really wouldn’t want to sit down there talking with a lot of people sitting there. [The sunroom lounge] is quite pleasant and in the sun it’s quite nice but if there are lot of people no. … there’s nothing you can do but I worry about some of these people [patients in the ward] who just sit here and don’t have anyone … cos there’s no nowhere really that you can go and sit sort of thing. Not that I would be in there. That little sunroom’s nice but you know it’s not really big or anything like that” 2656-02  “[The sunroom lounge is] where I turn that’s where you turn make a u-turn … I think there’s hardly ever anybody in there. I’m gonna to sit there on my own? I might as well come [to my bedroom] and sit in here. Yeah so I don’t use that much. If there was people in there then I would sort of stop and have a bit of a chat with them and say yeah how you going, but when there’s no one in there you think ah well I might as well head for home [to my bedroom]” 2656-04  “they could have more fun faces around here … Makes you happy … everything everything is empty today. That doesn’t help with the sick people. I feel I feel empty” 2656-05  “[The café and outdoor area is] a good thing to have there. Yeah because often visitors are um at a loose end. There might be something happening, so they’ve got something to do. …You know it's a nice place for people to come and sit, isn’t it.” 2656-06  “I’d definitely make a room a big enough room to hold enough people here that I wouldn’t say everyone would like to go there but some of them to go there … a homey room you know where everybody can have a little talk and be friendly and things like that. … I’m in this [bed]room all day. Pretty boring and I’m watching television. Pretty boring. You know what I mean? Until my children come in .. so pretty boring. It’s alright if you’re a sleeper, and I’m not a sleeper … I like talking. And you can’t in that [bed]room because I .. no one there … I like people.” 2656-08  “[When my children visit] we go downstairs. Down to um have a coffee. We go down and have a coffee and um if it’s raining we go in the, we still come in and there’s a little place down in the hospital here … it it’s just where you come in and just over this side you can sit down … and it’s very quiet. It’s very quiet yeah … and it’s got big seats. Yeah very nice. We sit there. … instead of sitting up there in that [bed]room … we sit here and we just talk to the people they go and get the lift. … the chairs are nice and colourful. You know it’s not dull. Is it? And ah people are coming and going for the lift. You know and sometimes they’ll have a little chit chat to you. You know and my son and daughter in law they like sitting here. They don’t like sitting in that [bed]room.” 2656-08  “I mean I guess it part of the the sunroom could be .. you know .. like you’ve got the TV there so if people don’t want to actually sit in your room and shut the door because or .. turn the TV on. If there’s someone else on .. you know sit down with other people. … [The sunroom lounge is] like not in a good position um but it’s not really .. very friendly, the books are everywhere and it’s not the shape of the room isn’t really good” 2656-18  “[I would like], not very many rooms but you know enough [rooms] to to for all the visitors, I mean the patients, to know all one another. To feel a little community there.” 2656-20 | “I think [the lounge room is] good. … I was thinking maybe it could be a little bit bigger but I mean that’s something because I have I guess a bigger family.” 2657-03  “I think this [lounge nook in hallway] is nice sort how it is. Yeah. But on the same token I wouldn’t come in here if anyone [else] was here. Even though if you met them through your group, I still wouldn’t. … I would say that’s just me. If I’m on my own I probably won’t go in there because they’ve probably already started something or they’re doing something and yeah I would just I would walk away. … Back to the [bed]room” 2657-03  “[The lounge is] quite a good space. Yeah ah they have books and TV there and DVDs and stuff so games and whatever so yes I’m not sure how much it’s utilized but yeah. But it was a good space to do [stroke morning tea] … there wasn’t people coming or going all the time or anything you know you had your own space yeah and it wasn’t threatening or anything, wasn’t medical apparatus everywhere” 2656-04  “Yeah downstairs [at the café] is good [when my wife comes] cos you can have a coffee and have a sit and talk yeah” 2656-04  “Come down [to the café with my daughter] and have a coffee. … We can talk to one another in private … well you're not crammed not crammed in a room. … [or] we just come and sit [in the garden out the front of the hospital] and yarn and talk.” 2656-05  “This [lounge room] here I like. This one here. Um, you can come in here and watch TV, get a book, um, they played bingo this morning. Um and the stroke people that’s where they bring you for morning tea and a chat … once a month that is, I’ve only been once … Oh [and] I’ve played bingo” 2657-06  “there’s a lot of other people lot of other people probably some people would look forward to have people to talk to … I do I do go through ah PT and talking with” 2657-07  “I need to have positive people round me. … Yeah. You need that.” 2657-12  “There’s plenty of seating around and I reckon that’s good. They have some good couple of rooms to entertain the kids and that as well so. When the kids do come they can go down there and play and that’s important.” 2657-14  “Maybe bring in some games systems in the common rooms and TVs and have some competitions between the patients. … In the common room. Haven’t they got common rooms here?” 2657-16  2.3.12 “comes in here come from long way and they come they can go down and have lunch in here. The other hospital you couldn’t do that. … And I tell you they enjoy it cos they have a rest. If I sleep they say well let’s go have something to eat and come back and see me” 2657-13 |

**Theme 4 - The environment should be legible and patient-centred**

| **Sub-theme** | **Case 1 quotes** | **Case 2 quotes** |
| --- | --- | --- |
| **4.1. (Dis)orientation in the physical environment** | 2.3.4 “the TV screen [by the nurses station with patient clinical information on it]. On that’s got admissions and you can often tell when there’s something coming for you [laughs] … whether you’re getting moved or whether you’re getting another medication or whether you’re out of the red or… I still haven’t worked it out. I used to have a bed monitor which is now gone. And that was in a red block. … Where my position is and am I coming up to getting out? Cos that’s where it gets indicated. It’s got the discharges and the dates. And that’s the only place you get true information. Cos you get half stories [from staff].” 2656-01  “[Looking out window] See that’s the gym there and they’re building more um retirement villages and things like that and the cafes just down there. … it’s interesting cos I was looking thinking well I was wondering whether that was Doncaster shopping town through over there [view] but they said no it’s not” 2656-02  “[I walk in a circle] right around the ward. Yes, I walk down the line and then you come to a dead end. Yeah that’s where I turn right and come back down or when I get here the other side of the desk I could either turn and come into here or I could go further, its only about 20 metres or so, and then you come to doors you can’t open so you make a youie [u-turn] [laughs] and come back to here. … [The sunroom] that’s where I turn that’s where you turn make a u-turn” 2656-04  “didn’t even know how many floors there were” 2656-06  “Oh yes so I know this is where I would have parked now [have walked out the back of the café and orientated herself] … Ah this is where we sat the other day. She had a coffee and I had just had lunch or something.” 2656-06  “I haven’t been anywhere else. I don’t think. Just the gym. and back here [to my bedroom].” 2656-10  “I just didn’t know [the ward lounge] was here. I didn’t know it was for the use of everybody.” 2656-10  “I was hallucinating and the nurse swore that I was in the [hospital] but I felt like I had been transported to my house in [suburb]. The door closing sounded exactly the same in fact she she even opened the blinds up so I could see that the room the same room. … I thought I was somewhere wrong .. somewhere else .. wondering how on earth I was out there without being noticed and then I calmed myself and looked at the ceiling and I realized that I was in the same place so I didn’t have anything to worry about … Because [the bedroom ceiling is] so distinctive. … yeah it’s saved me a couple of times from making a fool of myself.” 2656-10  “where they’ve got that green felt part near the café” 2656-11  2.1.24 “People need to know the fact that [the café is] available that there isn’t .. that it is there but they also need to say you know that if there was a like an information sheet you know beside your bed for information OK it says OK when it comes down to certain points like the cafeteria operating hours 8 til 5 and not open weekends um .. then people can plan around that you know. Um whereas we had to make up things as we went so yeah it um yeah but it was .. yeah it was ah not a big thing but it’s .. to me it all helps with patient wellbeing and patient um .. cos like I said before early in the piece without patients well if patients aren’t happ well if patients are happy it just makes makes it more difficult for staff because then they get grumpy right and they’re less cooperative um you know all sorts of things. So it’s .. yeah .. specially I think if patients were um .. given more information um and not have to wait around for information um ah it would be a lot better and there’s certain there’s certain areas, certain information that staff don’t have access to, they’ve got to wait for the doctors or physios or something like that, but there’s certain day to day running that patients can be given adequate information you know.” 2656-13  2.1.33 “the sunroom to me is the sign says sunroom .. oh yeah OK it’s solar, it’s a place where you get sunlight. It doesn’t say that there’s tea and coffee and anything like that and there is but it doesn’t say that and it was only because I sniff around [laughs]” 2656-13  “I don’t know it all looks the same” 2656-14  2.1.20 “[We] didn’t even know [the café] existed so that’s a problem. They need to tell people that. Maybe they’re worried that once people know they might wander down there themselves” 2656-15  “No I’m not up to [going anywhere else in the building] yet. The family have offered but they often bring me up drinks. But no I’m not up to that so I can’t .. I don’t know what the building even looks like on the outside. I just know the corridors, the lifts and that’s about it.” 2656-17  “This ward is good especially because when you not feeling that, um you know getting used to it and it is that circle … Yes and you can [find your way back], you know two or three laps or something” 2656-18 | “Well you’ve got a fantastic view from the [bedroom] windows … It’s like going in the gym room it’s got one big massive um window. … you could see the whole the whole town really you can you know and you don’t realize just how big Bendigo is until you look at it.” 2657-01  2.2.17 “I was saying downstairs I don’t know where they are (laughs) … I guess you know you get put in your wheelchair, you get wheeled around and you don’t know where you’re going. (Laughs).” 2657-02  “my next aim yeah is just to get out[side]. Probably get my bearings more so than anything” 2657-03  2.1.25 “have a little bit more information um about strokes. I know there are things there, but I just I don’t anything, maybe a little bit more information. Apparently they have morning teas and things down there, but I haven’t been or haven’t really known about it until too late to actually go, which that would probably be beneficial um but yes I haven’t been, I can’t say anything because I haven’t been to the morning tea down there.” 2657-03  “Bloody rabbit warren” 2657-05  “[besides my bedroom I go] to speech pathology and gym and that’s it” 2657-05  “Q: Okay. You know your way around. A: Yeah well. It’s been 5 weeks. You learn” 2657-06  “I walk around [the ward]. I walk around the 6[th floor ward] a bit but I don’t go anywhere.” 2657-07  “[I go] down to gym. And the toilet. Where else do I go? No, I’m going for breakfast one morning.” 2657-08  2.3.3 “If I had known that [ward lounge] was there I would have gone to it. But there’s nothing to say in here or anywhere that there is a place to go upstairs or wherever to get a cup of tea yourself, to get some magazines and books. No information at all. … Perhaps if that was advertise somewhere that’s simply and easy to read without having to go to a lot detail would have been nice. Cos I would enjoy going out.” 2657-09  “having a toilet sign inside does not help. The toilet it says, anyone could come from outside and go to it. … I didn’t realize it was a private toilet at the start because it was so public with the sign. … I found that very confusing. … it is a strange .. I don’t think it’s a good place for it because somebody coming from that direction could just wander through.” 2657-09  “I don’t go very far because at the present time I can’t walk that much you know and it’s driving me nuts to be honest. … It all looks the same to me. … How they get around those tunnels I’ll never know.” 2657-10  “[The physio took me to the outpatients gym] just to go down and have a look, show me where it is … I suppose to have a look around and familiarize myself with it.” 2657-14  “Oh the building? Bloody oath the place you can’t find your way from one end to the other … I don’t know how they find their way around it! Q: What do you think would help you find your way around it? A: Signs. No bullshit. Signs! … walked around the ward and got myself lost three times … it’s so easy bloody easy to get lost in here” 2657-15  “I had a couple more [pictures by my kids] on me door but I don’t know where they’re gone … Yeah [they were on] this side of the door so when the door was open and I used to come down the hallway and I knew which room was mine straightaway. Now I have a bit of trouble. I gotta wait til I get to the number and see the number. … I don’t know someone just took them down.” 2657-16 |
| **4.2. Convenience and suitability of the environment (fit-for-purpose)** | “the spacing of the drops [for the curtain] they got them really big on the ends but where the corner is, it’s too big cause one stays around that side of the corner and there’s one here, you try and pull this way it stops there R: so you can’t close the curtains D: it needs more things put in it so it can roll around. That’s just design.” 2656-01  “if you look look at this lead (points to lead connecting the bed controls to the bed) … See electrical tape wrapped around it? This was only done 3 weeks ago by electrical tape. That wouldn’t pass. … It’s electrical. It’s been pinched and it shouldn’t have tape around it, it should be pulled out of service.” 2656-01  “This [wheel]chair. It came off Noah’s ark. It’s a heap of rubbish everybody hates it here. The angle of yer feet. Look at me feet hanging over the end. If I push out it locks up, see? See? When I push back to come back … nobody knows what that bolt’s for. They got lots of sharp jagged edges on it. This cable – I got caught on the bed there and nearly tore off the thing … It was sticking out like that. It just dumb.” 2656-01  “this [bed]room is extremely hot. Everyone who comes it here says phew. So I leave that door open there [to the hallway] and that door open there [to the bathroom]. … Bit of breeze to come through. Otherwise I just wake up in a sweat. … My back actually feels wet on the bed, which is uncomfortable. I end up throwing everything off. So I wake up this morning with nothing covering me. … Every now and again I open the door [to the balcony] to let a breeze through but it flaps the curtain there. Flicks it if there’s too much coming through. … That [balcony] out there I think it’s a waste of space. Everybody says you’re lucky, but I don’t use it. See, if get out there with this, the [wheel]chair, I’ve got to really struggle to turn this around again. Not only that, it’s like a sauna. Glass, two walls, it just heats up.” 2656-01  “with some of the, the locks on the doors. So there’s a little lock … my sister’s been out buying stuff … it’s got all of these things and some of them are locked. … there was nothing here. There was no bowls there was no absolutely nothing. So [my sister] just went out and just went to coles and brought you know that plastic stuff. Which is fine, which is fine. Q: So I’m just going to explain that for the recorder, but just that there’s cupboards [in your ADL bedroom] that have signs on them saying plates, bowls, mugs, but they didn’t have any plates bowls or mugs in them. … A:And somebody came and said oh no all these things are here. Oh, no they’re not! …. if I’m going to do something I want to make sure that it’s right … Cooking. Or something like that.” 2656-02  “And that there was a problem with the sink wasn’t working, which I wasn’t, I wasn’t particularly worried about, but everybody else was … there’s water collecting so that could be potentially a problem … I don’t know and people have said they have reported it … the manager’s in the next morning, that was the weekend, Monday, the people were here and they said this has never been reported. … with the water and just sitting there … even if you were just lying here. In bed and if you’ve got water that’s just been collected from whatever, um for for you know for a week, now I mean I would never do that at home and I would never do that, but you know even here I think you know it’s far enough away from it, but that’s not good if people are not well and have um problems with their lungs and things like that” 2656-02  “The gym’s quite large and um they’ve got about 2 or 3 walkers or whatever you call them you call them something else … Treadmill type thing yeah and then a couple of bikes. I haven’t tried the bike, I’ve tried the treadmill. I’ve been using the treadmill um and then there were a couple of over arm things that I’ve done and I was doing those and there’s standing and doing your um getting your balance right … it’s just it’s big so there’s enough room for it. It’s got everything” 2656-02  “I suppose that if I was more motivated I would go over there and read [my timetable] but other people have read it and told me about it … I can’t possibly read it at this distance” 2656-03  “I mean have a look at the room we got three beds. Heaps of room. I mean you look probably in some other rooms and they might be a little bit smaller but not that much. Everybody’s got plenty of room in here so I think that ah if people are complaining to say aw you know it’s squeezy and that. Nah no there’s nothing I don’t think they have anything to complain about in that respect yeah. So when they brought me here I thought there would have been four beds yeah well I mean you look from that door there looking in, and I thought oh yeah it’s a four bed room, but when I walked in and I saw and there was only three I thought well even better. So there’s plenty of room. So I don’t think I would have had as much room if I had have gone to St Vincent’s [campus closer to the city centre] because their rehab was in the old part of the building that rooms were not great you know. You might say room is more at a premium … the position they’re at. You’re in the city so they gotta make every foot of space count.” 2656-04  “This is about the second week that I’m here and I’ve seen the physio come here once. … Never saw him again. … I walk on me own … If I want some sort of arm exercises you know to strengthen this arm here. I was the one who got this tennis ball. You know. … I’ve been doing this [squeezing the ball]. When I first come in here I could hardly move this [arm] like that. … And by doing that it builds [my arm] up, builds it up the whole thing. I think they’ve come to me and said who got you this? I’ve got it. Me daughter brought me this and she said here dad here’s a tennis ball for your hand. … if I hadn’t gotten this ball I would not be going home on Friday … And that was not because the physios, I mean the physio should’ve come down and said look here there’s this [ball] … a little bit slack on their part. You know you’re waiting and you’re waiting and you say aw yeah they’ll come today or they say I’ll see you tomorrow [laughs] but tomorrow never come” 2656-04  “the T.V doesn’t work … it comes on and off. It’s got its own mind it does what it pleases. Yeah if she wants to stay on the abc that where she stay nah I don’t want to look at it … Oh look I am interested yes but when you turn the T.V on you think what the hell it’s on SBS and it won’t go any further … it would be nice especially at night time … Well I mean during the day you’ve got other people walking in but during the night you sort of like the sound, and that’s when really you could say oh well maybe it’s interesting you know watching something like the old ones that’s on like Current Affair at 7 or 730. But I can’t get the bloody thing on there and if you do get it it’s all black you can’t recognise the people and you only hear the sounds … Oh I can’t be bothered [asking for it to be fixed]. You’re getting it for nothing. I mean St Vincent’s is the same. You get your TV for nothing. But in a lot of other hospitals you pay them between 8 and10$ that’s where I would be cheesed off if I hired them and it still wouldn’t work.” 2656-04  “the facilities mm .. not amazing (laughs). Could be better. The toilet I would say. I get into the toilet always find a .. another piece of toilet on top that toilet I don’t know what how to describe [participant is referring to a commode] but I had to take it out and put it away. And um the bathroom mm .. the one I’m going in, it’s a bit small. There’s another one further down but we can’t all go onto bath because the other people go in there so .. um it’s smaller than … you haven’t got where to hang your your clothes” 2656-05  “All this stuff [equipment in the hallways] … They’re all over the rooms. But on the other hand where [else] are they going to put them!?” 2656-05  2.2.24 “This equipment it’s remarkable isn’t it? [had just walked past equipment in the corridor] All of them. The things they have to help people. Yeah. Yeah … it looks to be so simple to use too.” 2656-06  “This is a great place here [in the gym], isn’t it? Yes, when they were discussing whether I’d go to here or to um [other rehab facility] yeah I said I’d like to be closer [which is the other rehab]. Yeah. But then um someone checked it out [here], and they said that this one had better equipment and everything … and to try and go here … there’s a lot of equipment here, yes. You know I can see what they mean then yes. And they’ve got room to put the equipment in haven’t they. … Is this part all new?” 2656-06  “it’s a good wide corridor. Yes great for this [walking frame] isn't it?” 2656-06  “I’m not up here [in the gym] for the view I’m up here to do the walking you know?” 2656-08  2.1.12 “There’s nothing I like [in my bedroom] Not even the bed .. it’s turned around the wrong way. [I’d have it] That way [pointing out into the room instead of against the wall]. Well I think it’s better than this way. I don’t think it should be this way. … they said I fell out of bed and I’ve got the wall to protect me … but they have paper down there [referring to a mat that goes on the floor by her bed when she’s asleep] under there somewhere yeah and they put that down and I haven’t fell out for a long, long time. And look how little the bed is. On the edge side. My bed [at home is] big. Look how little these beds are? Is it any wonder you fell out? Because you’d be turning over and you turn, move over and next thing you’re on the floor. The beds are to small and you can see like the width and they’re all new” 2656-08  2.2.25 “I like the gym down there and the bed that I’m sitting on … Cos it’s solid and easy to balance myself”2656-09  2.2.44 “we need more hoist for the building because we only have one here (coughs). We have one running running every room and the nurse is working hard with patient .. can’t give me anything for the patient and not to run after the hoist and this is really need to be changed .. very important and is .. can make the patient feel very low. Look at the nurse working harder then than what it should be and they really are tired you know. They need energy for the patient not for the hoist … They have more than more than enough thing to do because not .. I’m not the only one and also you know I think you can imagine when patient want to go to the toilet they have to go .. or waiting for the hoist to come and to take them to the toilets yeah that’s unfair you know. … and a a commode chair we need more .. they always run after the commode chair and people need to go to the toilet they have to waiting from time to time for when the other .. when one finish then the other can use it and usually you know .. they come and take it .. there was no one would remember to bring it back. Because there’s not enough. … Any how look like we don’t have enough commode chair. … [I feel] bad for asking .. yeah. It is. I do feel that. I feel bad for asking and I tend to wait and wait but you know you you only can hold your bowel to the limit time. Yeah. It’s very hard those .. hard feeling … yep. … But I don’t understand why it’s happen this way. It shouldn’t be that difficult to get these things to the building like this because it it’s .. we need it .. the patient need it not the nurse need it.” 2656-09  2.2.3 “having to ask permission to do everything [is frustrating]. And needing a staff member to to move the phone six inches or something .. very annoying. It’s just silly little things like that which I want to be back in my own house so I can .. if I needed the phone moves six inches I would think the problems through and find a solution whether it be a mechanical solution or .. calling someone to help me with it or whatever or just get a piece of stick and move the phone closer to me. Ah I’m not allowed to do any of that. No independent thinking and in all respects I’m [usually, prior to stroke] very independent.” 2656-10  2.2.26 “I’m not allowed to get out of bed by myself and I’m not allowed to stand because I’ll fall they say so there’s not much I can do. I can push against the the bed while I’m in bed. And I do a lot of that because I fall forward in the bed so I I lay the bed flat with the electrical thing .. device and then I’ll push against the baseboard and pull myself up towards the headboard and that’s the only exercise I can get. I do that quite regularly.” 2656-10  “maybe there shouldn’t be any carpet [in the hallway]. … it would be easier with the wheel chairs and tiled surfaces I would have done but then I’m not doing the pushing.” 2656-10  “The airconditioning seems to work down there [in the gym] where it doesn’t work that well upstairs [in the ward]. It’s always too hot. I suppose that the really sick people can’t regulate their temperatures. But um it’s very hot at night going to sleep but then it’s cold in the morning so .. I have no idea what’s going on there.” 2656-10  “I’d utilize [the gym] more. Need more people. More staff so that I can practice more .. but at the moment it seems like two steps forward and one step back. And I want to exercise.” 2656-10  “I need more ah bench space [in the bedroom] cos this [table] always fills up when a meal comes and then I’ve got to clear. A couple more of these [tables]” 2656-10  “these surfaces are very old. …[and] it’s an old toilet, single flush. They went out with button-up boots” 2656-12  “The waste. So much is thrown out. Food is an example. The trays just go out covered in food. Food could go into a big recycling.” 2656-12  2.3.26 “the room is not conducive to having more than one visitor cos there’s only one chair and the bed and um like on um what’s .. on the Saturday I had my two grandsons, my wife, um my daughter and her partner um and we came down here [to the loungeroom] … because there just simply is like not enough room. … if you got more than one visitor at a time there’s nowhere for them to sit except on my walker and if I didn’t have a walker, where would they sit? Particularly if you’ve got other people there having visitors and there’s no chairs left right … those people have come to see me and if I can’t accommodate them to a point where they’re comfortable being comfortable around in the environment then it defeats the purpose of the exercise of the visit. Um it seems to it seems to .. look in an ideal world you ‘d have you know one big room and six chairs but that’s not always practical I understand that and I get that and it’s not um it’s .. practicalities involved as well but um it’s not always practical to have that situation but at least room for one extra chair or something like would be probably um .. you know or the provision for one extra chair would be probably um a good chair although I don’t know how they’d do it not in the current not in the current set-up anyway.” 2656-13  “Ah [the hallways downstairs], they were wide. They were bright. They were airy. The colouring is open .. it wasn’t .. you know it was neutral but it was not dark. It wasn’t closed in um there was light what comes um you know um. I felt it was good. I felt comfortable walking down it. Yeah. I felt comfortable walking down it. … there was um maybe areas that I could have if I needed to grab onto grab hold of something. So you know um yeah no I thought it was good.” 2656-13  “the toilet facility that services room 4, I don’t see why there needs to be a door through to the next room, right. So my idea would be to block off that door and expand, like you’ve got a shower ah hand basin on one side and you’ve got a toilet on the other side and a passageway in the middle which is a thoroughfare. Two doors one there and one there which one goes to the toilet and one goes to the bathroom. I would incorporate into a large um ablution unit if you like where there was plenty of room to move around for patients .. particularly in a rehab situation – less trip hazards, less danger of things happening you know. It's ok for people that are mobile but people that are not mobile, we got people in there um that have had knee replacements you know ah .. obviously had difficulty getting around, they’re on crutches, they’re on walking frames um and the majority of people in rehab particularly are in that situation. I’d take the passageway out. Remodel the bathrooms. Plumbing essentially from a practicality but I’d do a bit of that stuff, I’ve done a bit of that sort of stuff. The practicalities of it would be .. without spending too much money, remove, fill that wall in along the .. between the um ward .. room 3 and room 4 right? Um open up that that ablutions area um and tile it all, put new tiles down .. they need to be relined .. it’s badly .. badly needed relining. Um you know resealing and waterproofing and things like that um .. they’re asking for trouble I reckon. In terms of like ongoing maintenance. … there is a toilet facility in room 3 which is just as bad. It needs to be refurbished. I was there this morning actually because the other one was occupied. … and I was surprised it was, to me it’s pretty bad I reckon but that’s my opinion.” 2656-13  “I’ve also noticed that there are other toilets scattered around and they are quite spacious but they also seem to be a dumping ground for various chairs and so that points to me to a lack of storage. … you’ve got two bathrooms side by side. One of them I used the other day and I had to clear um two shower chairs and a weighing chair out of the way before I could even have a shower. Now that means that they haven’t got a place to store them and people are just using that as an alternative so you know. … Just to go and have a shower I had to shift three things (laughs).” 2656-13  “They had these disposable cups and things like [that in the tea making station in the lounge room] which I hate. … they’re a bloody nuisance to get rid of for a start right. … was disappointing the little paper cup that’s sort of .. well not even paper it’s kind of plastic actually. … they’re not disposable. They’re not recyclable.” 2656-14  2.3.27 “sometimes J[patient] will have a few visitors and I haven’t been able to find a chair. Like at the start we sat on the bed but I find that a bit hard to sit on it. I need a back on my chair. Yeah you know even like chairs like that stack on top of each other in the corridor they just need something. You know it’s terrible having people just standing around … I get up and I give them my chair and I sit on the bed which I don’t like. Some times if you just stand .. you feel like you’re not staying .. do you know what I mean? You know you feel like you’re not being encouraged to stay. An in our case a lot of the people who are visiting have come a long way. Q: Yep so you wanna be accommodating? M: Yes.” 2656-15 wife  “[The nurse call-bell and TV remote] fall off. We all drop them. Everyone drops them though. … They probably need clamping somewhere to the bed or yeah you know that bar there you could reach there and just grab it. We just stick them in the draw now … [if I drop it] I can bend down but I can’t feel it.” 2656-15  2.2.39 “It has been a bit a bit difficult to charge the iPad. Like I’d have to put it over there, there’s no power points and he’s got a radio that we’re trying to recharge but we can’t” 2656-16 wife  “I wanted [the bed facing] this way so you can see the telly properly. Whereas it was against the wall because I had to sleep on my right side, looking outwards … they kept on saying a million times until it drove you mad it’s for your own good. It’s for your safety. … because I’ve got a good strong right side if I’m gonna go anywhere I’ll I’ll roll to the right and then roll right out of bed and they put that purple mattress on the floor next to you so you landed on that” 2656-16  “the rubbish bin on the end [of my table in my bedroom] needs to be about about eight inches wide and 12 inches deep. … The rubbish bin at the end should be big enough to hold every single [food] container that’s delivered” 2656-16  “this overnight bag which holds about a litre of urine, it’s quite big and heavy and they have to come and empty it a couple of times during the night and it keeps getting tangled around my legs, if there was a hanger on the underside of the table then they could just clip the bag on it and then it drained downwards without a problem. … You know they’ll empty it before I go down to the gym and sometimes they’ve got empty it while I’m at gym. And one day the gym instructor had to come back up here to get the plastic jug that they empty it into and I thought ooh that was a waste of his time. They need a plastic jug down there” 2656-16  “what I don’t like [in the gym] is as you come through the doors that we’re looking at there there’s a breeze blowing that feels like a quite a strong stiff breeze and it’s as cold as cold. The temperature down there is about 10 degrees lower than it should be. As if you’re going down there to warm up and relax your muscles it’s too cold to do that. I frequently ask the nurses is there a warm shirt in the wardrobe there and I ask them could I put it on before I .. before I go down there which is why I’ve got the second shirt on today. So it’s you know you can’t go down there and freeze to death. You know it’s just it’s too cold to be comfortable. They always say oh yeah it is cold down there so well you’ve got an air conditioning system down there, turn it up.” 2656-16  “It’s pretty busy [in the OT room]. Sometimes you go in there and there will be three other people doing the same session as you are. All doing upper limbs or something so I think .. yeah you have four people sitting around one table while it’s not not quite enough space really there for that. Sometimes they’ve gotta move chairs out of the room to get everyone in. … I find that sometimes it would be better if you were up a bit higher and the table was actually a bit lower so you’re looking down on whatever you’re doing more. … If you’re above something and looking down it’s it’s easier than doing puzzles and things when they’re they’re down a bit lower” 2656-16  “The lifts are obviously a problem at the moment and the fact that they have to close one down completely makes everything bank up.” 2656-16  “One thing though I really worried about was when they lost hot water for three days. I thought you’ll have a patient revolt if you don’t get that fixed. … just a hot shower is something that everyone enjoys. … Yeah. Cold shower. They said do you want to have a cold shower or no shower and I said no shower. They said that’s what most people have said. They said you can have a hot wash. So they went and got a basin full of hot water and went from there. … But it’s a pretty poor substitute for a hot shower.” 2656-16  “it’s nice walking on a timber floor [in the gym]. It’s quite nice walking on that floor too. Particularly if you can get a bit of speed up and it becomes easier to walk.” 2656-16  2.2.45 “They tell me to drink but how can I drink if my drink’s over there or if it’s anywhere that I can’t just do that [reaches for glass] and they don’t always think of that. They just say you haven’t drunk your drink but I think well could i? And I do say to them how could I. Some of them especially one of the nurses is marvelous. He kept coming and topping up and make sure that the drink was there. Otherwise they don’t think of it. I find I have to sometimes and perhaps I’m just getting old and very demanding .. I hope I’m not but I often have to remind the staff to do this or that or say well while you’re there why don’t you grab such and such and sometimes I think they probably find it helpful and sometimes they I think I’m a damn nuisance. I don’t know. I try not to be a nuisance. … if I think someone’s going to come in I leave it til then and I say tick tick tick [have a list] … Yes well I like to feel as if I have still little bit of control. But it’s very limited I know. … Q: Are there other things that help you to make the most of the control that you do have? A: Yes yes the bell I won’t go anywhere without it. And to me it’s like at the moment, it’s very important with that bell. Try not to use it but at least it’s there and I don’t feel .. and I’m not saying, alone is not the word I don’t know the word but if it’s there I feel as if I have control. Q: Yeah. Yeah so are you saying alone is not the word? Do you mean because you don’t feel lonely is that right? A: No I don’t feel lonely. I just feel connection.” 2656-17  2.3.28 “I think very comfortable [having visitors in this room]. They always seem to move their chairs around. That’s all I can say. Very comfortable I feel.” 2656-17  2.2.28 “The good parts [of being in an ADL room with kitchen] are um you know being able to make a sandwich whenever I feel like I have. um Coffee when you want it. Hot toast (laughs) instead of cold. Those things are the good things. … And it’s not just about that it is nice to have a coffee or toast. It’s you you sort of have to remind yourself that this .. the reason is to get used to doing things .. liking I’m doing myself um medications and doing that sort of thing and all that. I think. Preparing for home so ..” 2656-18  “It’s good to I like that there’s a bright light but there’s also one here [next to my bed] that’s just .. so especially if I’m going going to the toilet or something and it’s in the middle of night” 2656-18  “So sometimes with the physio I’ll just have a sit there [downstairs near cafe] or do a bit of walking out there [in the therapy garden] on the different surfaces.” 2656-18  2.2.40 “[my bedroom is] not very convenient … Well look at this [nurse call bell cord] it’s attached way over there and it falls on the floor. That’s why I keep it in [the draw] because that’s not going anywhere … It’s not very ah oh it’s not very convenient for any of the electrical things. And you see I’m not allowed to stand up without the walker, so ah I have to if I want to charge anything I have to get that walker out or if I want to ah put my hearing aids on to charge overnight, which I must, otherwise I can’t hear anything the next day I have to get out get my walker go over there and sort it out um it’s just not conveniently what’s the word? Arranged. Or it’s not ah people ah there’s a word for it you know ah helpful. Helpful will do but that’s not the word … Oh no it doesn’t doesn’t make me upset or or ah annoyed with them or but when it falls on the floor I do say shit (laughs) … It’s just difficult getting getting to things like with the phone sometimes it take four or five or four times of rings before I can possibly get to it and by that time it’s stopped ringing here and it’s gone through to the switchboard so so knowing of course that it will be family or something and they will ring back again I just wait for a few minutes and they do ring through again. But it’s not nothing is conveniently placed … I can’t really find a word for it ah doesn’t make me angry, it makes me disappointed ah a little bit of angst like how am I going to get over there in time that sort of thing but it’s just a matter of placing” 2656-19  “I could say that there are not enough mirrors in [the gym] because while I was in Phnom Penh hospital they were just there … lots of glass like a ballet [studio] … people who can’t lift up their, can’t walk it’s you look to see the mirror better than right now is that you can use as a cue .. but ah there’s no mirror reflections giving me an idea of how I look walking” 2656-20 | “I mean I see they got this picture of someone who’s had a stroke [showing how she should position her affected arm on a pillow] but I can’t get over there to really see it” 2657-01  2.2.5 “I’ve got a cleaning lady that chats to me in the morning and she does 26 rooms and shower rooms all that cleaning. Elderly lady and she’s very nice and she give me a rubbish bags [taped to her table] so that I don’t have to worry and I thought that that’s lovely … she’s doing it for everybody giving them an empty bag because she said the bags are only little and so it’s worth it. So she saves all the shopping bags see and then makes for the cleaning. Makes it easy” 2657-01  2.2.15 “I put down that the nurses must assist with meal, but they don’t .. they just leave them. See I was got a fork upside down managed to get the milk out and stabbed it so I could get meself a cup of tea and then there was a lovely little box of .. just a little pack of fruit but I can’t open the .. so there I was bouncing it about, turning it over … There was a lovely little roll in a bag. And it was lovely and warm and there’s butter there and jam. I can’t do none of that. And so there I was with the roll on me beating the hell out of this paper bag well it was a plastic bag which would not open. Trying to stab it! [laughs]” 2657-01  “the first day I went [to the gym] was quite a few people sitting [in the waiting area in the hallway] in their wheelchairs waiting for their turn.” 2656-01  2.2.29 “you had these these things rails yeah and when I was up here in the other room .. the end room um they couldn’t find the trolley to take me to the toilet so they put me in these sort of straps and they sort of swung me along (laughs) .. I thought my god I’m going through the roof! … The hoist that’s what which is marvelous especially if you’ve got a customer or person that might be a bit heavy or awkward for the girls to push around but the machines like the what you’d put into the toilet. You seen these big machines .. They call is a steady … if you can’t pull yourself up and they put like the flaps down and you sit on that til they get you round to the toilet. Oh well I didn’t like that when I did the hoist but the other machine [the] steady, but the blue one is the one they use mostly and then when I come back from the toilet they sit me on me chair see and I just have to put me feet on it um … Well it’s easier. It is easy. Actually I’ve got more strength in my right arm than I realized. If only I can get this one [left] going.” 2657-01  “I think we that we can use that [gym] facility a little bit more over the weekend. It would be a tendency to get you out of the room, and you know into the normal regime of things really, what you do during the week.” 2657-03  1.1.13. “these hallways I think they’re great because they’re open. So you can actually see down to the other end. See a bit further really than what you want to go, which is good. … [you can look at the distance and think to yourself] okay right well I’ve got all that space and yeah I can handle it. … it’s great because it just feels like opened and there seems to be so much room um to be able to get around whether you’re requiring an aid to get around um I just think it would be a whole lot easier to manage in this particular circumstance, yeah, being open and so wide.” 2657-03  “It’s quite warm in [my bedroom] on a hot day … it’s the hottest room in the hospital … It’s a lot cooler [in the gym]” 2657-05  “[The garden out the front of the hospital is] where the physio took me. Cos it’s uneven surfaces … he took me down here and then we crossed the pedestrian crossing there, cross the other one, and walked up the hill … it’s all different um textures. And that was for balance” 2657-06  “probably make [the gym] bigger make it just a little bit bigger cause the other day there was one two three four five six seven eight there was ten of us. And um yeah that was in our group session the single sessions but your never on your own even in a single session you’ve got your one on one worker but you might have three or four others as well getting there. I still think it could be half as big again even for the staff that are coming there give them more space that’s about the only thing I would change I think because it’s fantastic.” 2657-06  2.3.24 “they have everything set up for us [in the breakfast group room] they have the TV going. Anyone in wheelchairs are they shift the chairs and put the wheelchair under. And it’s fantastic, it really is fantastic. … And they always the the papers on the on the table for the men who want to read the papers. No. It is really really. This is what I found was fantastic. And we all look forward to it.” 2657-06  “if I want to read at night .. a light .. I can’t see in the dark or reach up for a light” 2657-08  “You have to allow for trolleys and have allow for ambulance men rushing up and down so you have to that space which you might say oh that’s a waste of space but you need someone there to turn a trolley or something .. you’ve got to have it … [in the hallway] and these little [bed]rooms … And even that thing they put me on that stand up thing [the steady], it all takes space and you have to allow for that.” 2657-09  2.2.21 “I’ve been taken for a run round the garden and had a look at the helipad. My son did that. Yeah and if there .. there’s a wheelchair usually available for everybody to do that with their families.” 2657-09  “[the gym] in my opinion desperately in need of more space cos everybody’s getting around each other. You’ve got leads running everywhere … it’s not them it’s just the way it has to be. … it’s unnecessarily crowded because of all the things they have to have there … [and the gym is] not very well lit. … Depressing. Because people are trying to get better. They’re trying to do better, they’re looking at their feet and they’re being told to look up, look up, do this, do this and you have to follow instructions of course but um you get an old man in a wheelchair, another man learning to walk over here and there’s people running around to get their stuff and their gear and move things it’s quite quite busy. That was one of my first impressions of going down there. … Oh yes around here [in the gym] before they had a loose cord … it’s a danger to people you get your foot tangled in it or something especially those that aren’t walking well” 2657-09  2.2.1 “Having the timetable, the way they wanted to do it or had planned to have what appointments I have with physio and doctors or when the doctors are coming which is a big thing um and when visitors are coming in or when you’re expecting them and things like that .. something to look forward and then you’re disappointed if they don’t come (laughs). … I do [like having the timetable]. In fact I get cross because I can’t read it without my glasses. And I can’t reach me glasses easily.” 2657-09  2.2.20 “during the day people remember to pull it down if it’s too bright but at the night time they tend to forget about it and it’s really cold coming in there. It is. I have not been cold .. I have not been so cold in hospital ever before as I have been here and I had a blanket .. you know they leave a blanket and stuff but ah the blankets are only cotton ones which is good it’s easy to clean but you find yourself catching it and putting it on if you freeze (laughs).” 2657-09  “Yeah I like the gym it’s a good gym. The walking things with the mirrors … I was looking in the mirrors and when I seen meself walking in the mirrors that’s when I started crying because I could walk. I was seeing where I was putting me feet. Whereas before I couldn’t see where I was putting me feet so I couldn’t walk. … No it’s good.” 2657-12  “I’ve got to across to the outpatients’ gym and they got heaps more machinery and different weights. Our gym’s a bit boring.” 2657-14  “Hard floors everywhere. There’s no carpet in this joint at all is there? It all seems to be hard floors. That’s another thing I might add is a bit of carpet now and again in some places. Makes the floor a bit softer for people to walk on.” 2657-16  2.2.23 “I hate the TV. Touch screen is ah .. some places you gotta put your birth date into it to make it operate. It keeps happening quite often. Got to sit up in order to do it. And then it keeps doing it .. only just put it in it and then later same thing your birthday or your passcode .. and I’ve got to start all over again. Makes me quite angry I wanna punch it. Already punched one and broke it so then if I broke this one they’d never give me another one. I use it as much as I can but like I said it frustrates the crap out of me. Normally I’ll buzz a nurse and get a nurse to do it for me so I don’t break it cos I know if I keep trying I’m gonna get angry and I will punch it.” 2657-16 |
