# Supplementary material for: The role of the physical environment in stroke recovery: Evidence-based design principles from a mixed-methods multiple case study
Source: PLoS One. 2023 Jun 9;18(6):e0280690. doi: 10.1371/journal.pone.0280690 (PMC10256226; doi:10.1371/journal.pone.0280690)
Supplement: S1 Fig — (DOCX) [file pone.0280690.s003.docx]

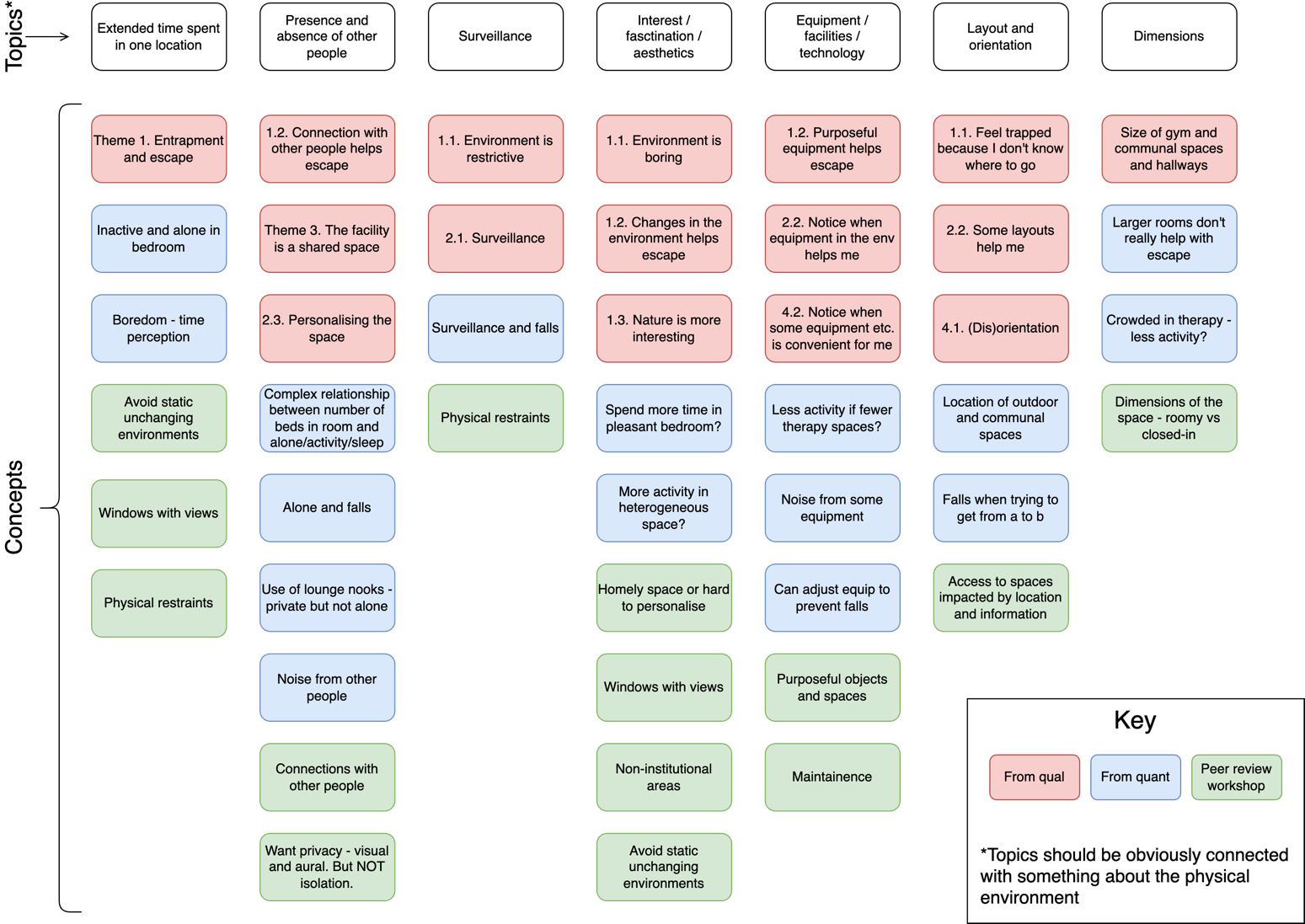


**S1 Fig. Topics describing the important aspects of the qualitative and quantitative findings.** The contents of the joint display tables used for the convergent mixed-methods analysis were organised according to these topics which were drawn from important aspects of the qualitative and quantitative findings. The topics were chosen during and after the peer review workshop. To choose these topics, the research team considered the qualitative and quantitative findings closely, identified concepts relating to the environment that appeared in these findings, and organised these concepts into seven representative topics.
