# Supplementary material for: The role of the physical environment in stroke recovery: Evidence-based design principles from a mixed-methods multiple case study
Source: PLoS One. 2023 Jun 9;18(6):e0280690. doi: 10.1371/journal.pone.0280690 (PMC10256226; doi:10.1371/journal.pone.0280690)
Supplement: S1 File — (DOCX) [file pone.0280690.s004.docx]

**S1 File. Healthcare Design Checklists used in the ENVIRONS study: Methods & Results.**

**S1. 1. Methods**

Evidence-based healthcare design checklists were sought to quantify the nature and quality of the physical environment at both case facilities. An extensive search of the literature and healthcare design websites revealed no checklists specific to rehabilitation facilities. Correspondence with the authors of a recent systematic review on instruments for assessing the quality of healthcare environments helped to inform which checklists would be most appropriate for the study (Elf et al., 2017). Two healthcare building design checklists were chosen: The Achieving Excellence Design Evaluation Toolkit (AEDET Evolution) and the Staff and Patient Environment Calibration Toolkit (ASPECT). These two checklists were chosen for several reasons. Firstly, they were developed by reputable organisations and are purportedly based on evidence from research. Both checklists were developed by the same collaboration between the National Health Service (NHS) in the UK, Sheffield University, and others (DH Estates & Facilities, 2008a, 2008b). The checklists are designed to complement each other (see below), which is why both were included. Secondly, they have previously been used in healthcare environments research (Abbas & Ghazali, 2011; Ghazali & Abbas, 2012). And lastly, while they are not specific to rehabilitation, each section can be weighted to reflect the relative importance for the specific healthcare setting in question.

The AEDET Evolution is designed to evaluate the design quality of healthcare facilities. It has ten sections: 1) character and innovation, 2) form and materials, 3) staff and patient environment, 4) urban and social integration, 5) performance, 6) engineering 7) construction, 8) use, 9) access, and 10) space. The three sections relating to engineering and construction quality (i.e., building performance, building engineering, and construction) were not completed as they were outside the scope of this study. Each section is comprised of a series of items which can be answered using a six-point scale, between 1 (virtually no agreement) and 6 (virtually total agreement), or the auditor can select a score of 0, which indicates that they cannot determine the rating for this item. The final score for each section is calculated by averaging the score of the items for that section. Items given a score of 0 are excluded from the averaging.

The ASPECT is designed to expand on section 3 of the AEDET Evolution by providing a more comprehensive evaluation of staff and patient environments. It has eight sections: 1) privacy, company and dignity, 2) views, 3) nature and outdoors, 4) comfort and control, 5) legibility of place, 6) interior appearance, 7) facilities, and 8) staff areas. The scoring system is identical to the AEDET Evolution.

To ensure familiarity with the building, the checklists were completed at the end of data collection period by the first author. Some sections of the checklists required input from staff.

**S1. 2. Results and cross-case comparison**

The results of the Achieving Excellence Design Evaluation Toolkit (AEDET Evolution) and the Staff and Patient Environment Calibration Toolkit (ASPECT) are summarised in Table B1 and Table B2 respectively. Items with less than or equal to one score difference between the cases are highlighted in bold.

Table S1.1. Results of the Achieving Excellence Design Evaluation Toolkit at both cases.

| **Item** | **Case 1** | **Case 2** |
| --- | --- | --- |
| **Character and innovation** | 2.0 | 4.8 |
| **Form and materials** | 3.8 | 5.3 |
| **Staff and patient environment** | 2.7 | 4.2 |
| ***Urban and social integration*** | *4.5* | *5.0* |
| **Performance** | -- | -- |
| **Engineering** | -- | -- |
| **Construction** | -- | -- |
| **Use** | 3.3 | 4.7 |
| ***Access*** | *4.4* | *5.0* |
| **Space** | 3.5 | 4.7 |

Items with ≤ 1 score difference between cases are italicised.

Minimum score = 1 (virtually no agreement). Maximum score = 6 (virtually total agreement).

Table S1.2. Results of the Staff and Patient Environment Calibration Toolkit at both cases.

| **Item** | **Case 1** | **Case 2** |
| --- | --- | --- |
| **Privacy, company, and dignity** | 3.7 | 4.8 |
| **Views** | 4.4 | 5.6 |
| ***Nature and outdoors*** | *3.3* | *4.3* |
| **Comfort and control** | 2.8 | 4.0 |
| ***Legibility of place*** | *4.2* | *4.5* |
| ***Interior appearance*** | *3.4* | *4.3* |
| **Facilities** | 2.3 | 3.8 |
| **Staff** | 3.7 | 5.3 |

Items with ≤ 1 score difference between cases are italicised.

Minimum score = 1 (virtually no agreement). Maximum score = 6 (virtually total agreement).

Although the cases received similar scores for some items on the checklists, the newer hospital, Case 2 (BH) scored consistently higher than Case 1 (SG) on all items in both checklists. The category of ‘Character and innovation’ on the AEDET received the biggest difference in scores between the two cases (difference of 2.8). This category deals with the overall feeling of the building and the clarity of design intention. It is perhaps unsurprising that the new building (Case 2) scored higher in this category than the older building (Case 1), as the older building is made up of many different parts and has been renovated a number of times, which has diluted any comprehensive design intention. In this way, Case 1 is likely representative of many of the older rehabilitation facilities in Victoria (Lipson-Smith et al., 2020).

The category with the next biggest difference in scores between the two cases was the ‘Staff’ category on the ASPECT (difference of 1.6). This category addresses the question of whether staff are well provided for by the building, e.g., whether there are spaces for staff on and off the ward. There were very few staff spaces at Case 1 (SG), whereas at Case 2 (BH) there were two nurses’ stations, a communal office, a meeting room, and an off-ward staff room and changing area. The lack of staff offices on the ward at Case 1 (SG) meant that most staff congregated at the nurses’ station. This gave a communal atmosphere to the ward but had implications for patient privacy as personal conversations and clinical handovers were carried out in public. Staff at Case 1 (SG) often sat in the patient lounge room or the meeting room on the ward while on their break, while staff at Case 2 (BH) used the off-ward staff lounge.

The categories that received most similar scores between the two cases were ‘Urban and social integration’, ‘Access’, ‘Nature and outdoors’, ‘Legibility of place, and ‘Interior appearance’. Although the cases scored similarly for these categories, the reasons for their respective scores differed. For example, the ‘Access’ category addresses the question of access to the vicinity of the building (including public transport and parking), but also addresses the question of routes to get in and out of the building once there (including ramps, lighting, external signage, and emergency access). Case 1 (SG) scored higher on the items regarding public transport and parking, while Case 2 (BH) scored higher on items regarding ramps, lighting, external signage, and emergency access. These scores reflect the location of Case 1 (SG), in a connected metropolitan suburb, and the new, coherent design of Case 2 (BH), with a prominent entrance to the hospital and up-to-date compliance with accessibility standards. In the ‘Interior appearance’ category, Case 1 (SG) scored higher on items addressing whether patients can display personal items in their own space (because the Public Private Partnership regulations at Case 2 (BH) made this more difficult for patients), whereas Case 2 (BH) scored higher on items addressing whether the interior looks clean, tidy, and cared for (the older interiors at Case 1 were harder to keep looking clean and well-maintained).

**S1. References**

Abbas, M. Y., & Ghazali, R. (2011). Physical environment: The major determinant towards the creation of a healing environment? *Procedia Social and Behavioral Sciences, 30*(1), 1951. <https://doi.org/10.1016/j.sbspro.2011.10.379>

DH Estates & Facilities. (2008a). Achieving Excellence Design Evaluation Toolkit (AEDET Evolution) Summary. Retrieved November 2017, from NHS <https://webarchive.nationalarchives.gov.uk/20130123193051/http://www.dh.gov.uk/en/Publicationsandstatistics/Publications/PublicationsPolicyAndGuidance/DH_082089>

DH Estates & Facilities. (2008b). A Staff and Patient Environment Calibration Toolkit (ASPECT) Summary. Retrieved November 2017, from NHS <https://webarchive.nationalarchives.gov.uk/20130123193049/http://www.dh.gov.uk/en/Publicationsandstatistics/Publications/PublicationsPolicyAndGuidance/DH_082087>

Elf, M., Nordin, S., Wijk, H., & McKee, K. (2017). A systematic review of the psychometric properties of instruments for assessing the quality of the physical environment in healthcare. *Journal of Advanced Nursing* <https://doi.org/10.1111/jan.13281>

Ghazali, R., & Abbas, M. Y. (2012). Assessment of healing environment in paediatric wards. *Procedia - Social and Behavioral Sciences, 38*, 149-159. <https://doi.org/10.1016/j.sbspro.2012.03.335>

Lipson-Smith, R., Zeeman, H., & Bernhardt, J. (2020). What’s in a building? A descriptive survey of adult inpatient rehabilitation facility buildings in Victoria, Australia. *Archives of Rehabilitation Research and Clinical Translation* <https://doi.org/10.1016/j.arrct.2020.100040>
