# Supplementary material for: The role of the physical environment in stroke recovery: Evidence-based design principles from a mixed-methods multiple case study
Source: PLoS One. 2023 Jun 9;18(6):e0280690. doi: 10.1371/journal.pone.0280690 (PMC10256226; doi:10.1371/journal.pone.0280690)
Supplement: S2 File — (DOCX) [file pone.0280690.s005.docx]

**S2 File. Semi-structured walk-through interview used with stroke survivors in the ENVIRONS study**

***Instructions for interviewer***

- The content and route of this walk-through semi-structured interview should be largely guided by the participant, not the interviewer. However, participants can be encouraged to access each of the following standard spaces: the patient’s bedroom/bathroom; any shared dining/recreation spaces including kitchenette; any outdoor therapy and/or recreation space/s; therapy spaces including the gym, ADL rooms, and consult rooms; meeting spaces; and common areas of the facility including entryway, reception desks, and corridors.
- The role of the interviewer is to ensure that all of the participants’ ideas/opinions/thoughts are explored in full.
- The interviewer can use prompts to encourage the participant to explore an idea further or to consider a new avenue.
  - A list possible prompts are included below.
  - These should be used to encourage participants to address the criteria that are considered important in the design of inpatient stroke rehabilitation facilities.
  - However, prompts should be tailored to the participant and based on their activity and the locations in which it took place during behavioural mapping.
  - You will probably need to use other prompts/questions in addition to those listed below in order to respond appropriately to participants.
- You can encourage the participant to draw/take photos to expand on their ideas.

***Preamble read by researcher to introduce the interview to the participant***

- Thank you for agreeing to complete this interview.
- The purpose of the interview is to understand how you experience the physical environment of the building that we are in.
- During the interview we will move around the rehab ward, taking any route that you choose.
  - You can move around the ward in any way you choose e.g. walking, wheelchair, walking frame, and/or frequent breaks to sit down.
- The interview will take about 1 hour, but the time will depend a bit on your answers.
  - You can take as many breaks as you like during the interview.
  - You can also stop the interview at any time if you want to. You can stop the interview for any reason or no reason.
- When we start the interview, please take me (the interviewer) on a tour of the inpatient rehabilitation ward.
  - Imagine that you are showing the building to someone who doesn’t know the space, and who wants to know what you do in the building, where you do these things, why you do them in these places, and how the different parts of the building make you feel.
  - You can talk about things that you like, and things that you don’t like.
  - Where you go on the tour is up to you – just go to all of the places that you spend time, that you have an opinion about, or that you think are important.
  - If it helps you to express what you think, then you can draw pictures or take photographs of parts of the environment that are important to you. **Please do not include any people or things that might identify a person in your photographs.**
  - The researcher will also draw a map of where you decide to go on the tour.
- With your permission, the interview will be audio-recorded so that the researchers can listen back to the recording later when they are putting together the results of this research. ***[Start audio-recording.]***

***To understand patient’s overall experience***

- Why did you bring me to this space/room?
- Why did you take that photograph/draw that drawing?
- Is there anything about this space/room that is important to you?
  - Is there anything about this space/room that you like/dislike?
  - Is there anything in this space/room that you want to take a photo of?
- Overall, what does this space/room (or ward) feel like to you?
  - What other type (or types) of building or spaces does it remind you of? Why?
  - How does this space/room (or ward) make you feel?
- What do you do in the evenings and weekends in this space? How is this different from what you do during the day on a weekday?
- If you were talking to your daughter/friend/husband on the phone, how would you describe the design or environment of this space/room (or ward)?
- If you could change anything about the space/room (or ward) what would you change? What would you not want changed about the space?
  - Have you spent time in other rehab facilities before? Or other hospitals? How does this one compare?

***To encourage visiting more spaces during the walk-through interview***

- Are there any other places in the ward (or building) that you would like to take me/that you would show to someone who has never been to this place before?
- Yesterday/the day before yesterday, you did [activity/inactivity] in [room/space]. Is this somewhere you would like to take me?

***General probes to elicit more information***

Taken from Gillham, B. (2005).

- Clarifying: I don’t quite understand that, can you explain it to me?
- Showing understanding: How did you feel about that?
- Justifying: What makes you say that?
- Relevance: You’ve lost me, how do those two things connect?
- Asking for an example: What do you mean by…? Can you give me an example?
- Extending the narrative: Tell me more about that.
- Accuracy: Now, let me see if I’ve got things in the right order?

***Specific probes to elicit more information***

Practice of physical and cognitive (inc. social) functions:

- What do you do when you’re here?
  - Yesterday/the day before yesterday, you did [activity/inactivity] in this space.
- Why do you/don’t you do these things here?
- Do you think this space provides you with opportunities to practice physical activities? Or to do activities that require thinking? Or to socialise?
  - Apart from your scheduled therapy, what do you think your opportunities are for getting practice doing physical things, things that involve thinking, or socialising?
  - What do you think influences the amount of practice that you get (this could be something about the built environment, or it could be something else)?
- Does the space make you feel motivated to practice doing physical things, things that involve thinking, or socialising?
  - Where do you feel most motivated? Why?
  - What sorts of spaces help you to feel motivated? Why?
  - What do you think most motivates you (this could be something about the built environment, or it could be something else)?

Sleep and rest:

- Tell me about how your sleep and rest has been since you have been on this ward?

Emotional well-being:

- How do you think this space interacts with your own emotional well-being?
  - How does this space/room make you feel?
  - How do you think this space/room interacts with your mood?
- How do you think this space interacts with the emotional well-being of staff (i.e. how they feel in themselves)?
- How do you think this space interacts with the emotional well-being of family family/friends/visitors (i.e. how they feel in themselves)?

Safety

- Do you feel safe here?
- How do you think this space/room interacts with or impacts your safety?
- How do you think this space interacts with or impacts the safety of staff?
- How do you think this space interacts with or impacts the safety of family/friends/visitors?

Other criteria in the framework:

- Is there any technology in this space/room that you think is important? What is it used for?
- Can this space/room be used for more than one thing/task/activity? Is it used for more than one thing?
  - What about the corridors/foyers? What do you use them for? Are they used for more than one thing?
- Do you feel like you have control over what is in this space/how it looks/what you use it for?
- Do you have access to outdoor/green space? Do you use it? Why/why not?
- Do you feel like this facility is integrated with the wider community/outside world? Why/why not?
- Any comments about noise/smells/air quality/light?
- Are places in this building hard or easy to find? Did you have trouble finding rooms/spaces or understanding what they are for?
- Are there any spaces that are ‘off-limits’/that you can’t get in to/can’t access without permission? Why?

***Summary at end of interview***

- *[Interviewer provides summary of what was discussed.]*
- Do you have any other comments about the design or environment of this ward?
- Would you like an opportunity to review an audio-recording, transcript, or summary of your interview before it is analysed?

*Interviewer thanks participant for their time and stops audio-recording.*

**References**

Gillham, B. (2005). *Research Interviewing: The range of techniques: A practical guide*. McGraw-Hill Education (UK).
