# Supplementary figures and images for: The role of the physical environment in stroke recovery: Evidence-based design principles from a mixed-methods multiple case study
Source: PLoS One. 2023 Jun 9;18(6):e0280690. doi: 10.1371/journal.pone.0280690 (PMC10256226; doi:10.1371/journal.pone.0280690)

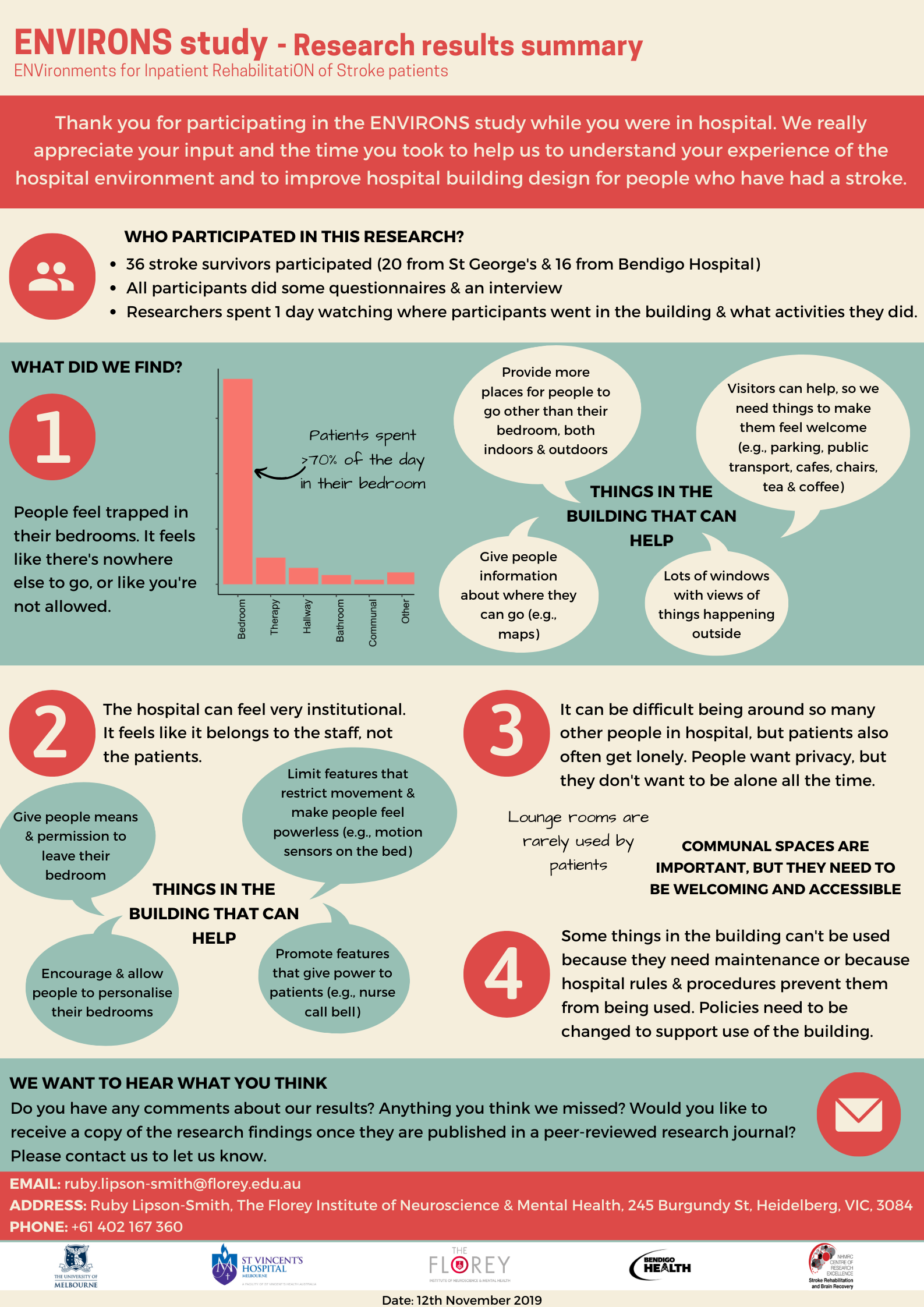

Supplement: S3 File — (PNG) [file pone.0280690.s006.png]
