# Supplementary material for: The role of the physical environment in stroke recovery: Evidence-based design principles from a mixed-methods multiple case study
Source: PLoS One. 2023 Jun 9;18(6):e0280690. doi: 10.1371/journal.pone.0280690 (PMC10256226; doi:10.1371/journal.pone.0280690)
Supplement: S4 File — (DOCX) [file pone.0280690.s007.docx]

**S4 File. Further quantitative findings from the ENVIRONS Study.**

*S4. 1. Further findings from the behavioural mapping regarding participants’ activity and time alone in single and shared bedrooms*

The time spent alone in the bedroom was further interrogated by considering single and shared bedrooms at each case. Figure S4.1 shows the median number of observations that participants in single and shared bedrooms spent alone while they were in their bedroom. At both cases, participants in single-bed rooms appeared to spend more time alone than those in shared bedrooms, but the difference between single and shared bedrooms appeared to be less pronounced at Case 2 compared to Case 1 (see Figure S4.1). One reason for the smaller difference between amount of time alone in single and shared bedrooms at Case 1 could be that all of the participants who were in a shared room at this case were in a three- or four-bed room, and so would not have been left alone if one roommate was absent, while many of the participants who were in a shared bedroom at Case 2 were in a two-bed room and so would have been alone if their one roommate was absent (see Table 4 in main manuscript).


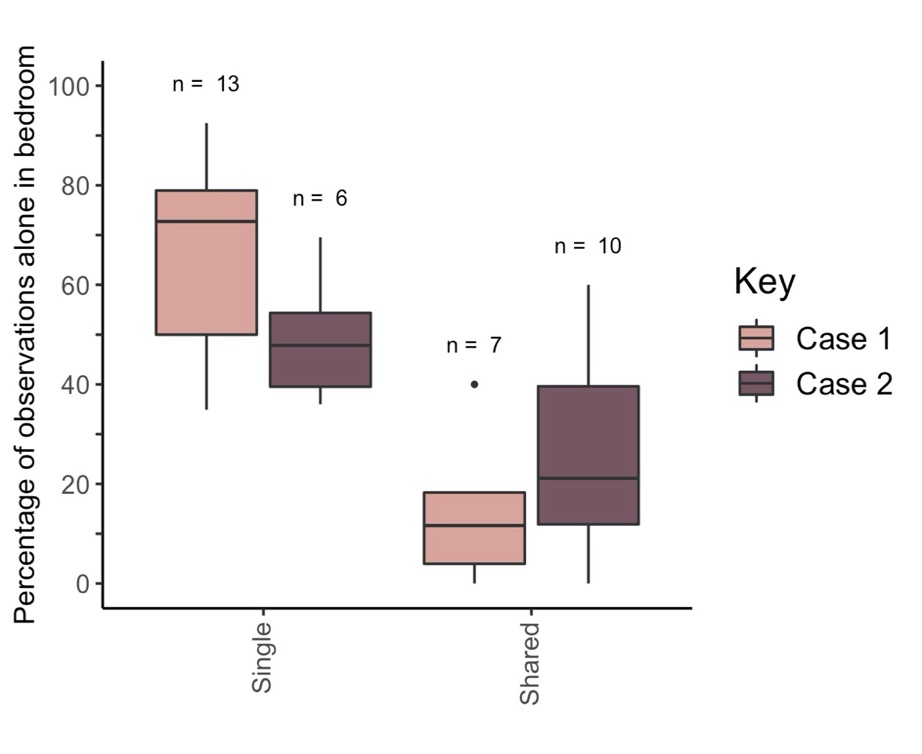

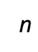

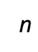

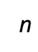

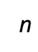


Figure S4.1. Median percentage of observations spent alone in single and shared bedrooms by all participants at each case. The number of observations that participants were alone as a percentage of the total number of observations that they spent in their bedroom. Boxes indicate median and interquartile range. Dots represent outliers. Single = single-bed room, Shared = shared bedroom.

In addition, the participants at Case 2 may have been more likely to have been categorised as alone in their bedroom, even when a roommate was present. A participant was considered ‘alone’ if there were no other people in the vicinity capable of social interaction. People who were asleep, severely cognitively impaired, or behind a curtain were therefore not included. Some of the participants at Case 2 shared a room with patients who had been admitted under a different classification, i.e., for complex medical care rather than for rehabilitation (see Appendix A). Participants sharing a room with one of these patients would have been classified as ‘alone’ if their roommate was asleep or too cognitively impaired to communicate. What is more, field notes indicate that the retractable dividing curtains were often closed between the beds in the shared bedrooms at Case 2, which also would have resulted in a participant being categorised as ‘alone’. The dividing curtains were more substantial and easier to use at Case 2 compared to the curtains at Case 1. These results suggest that there are many variables in the physical environment of the bedroom which may influence how much time patients spend in there alone, including number of beds, roommates’ impairments, type of barriers/curtains between beds, and position relative to communal hubs, to name a few.

Figure S4.2 shows the percentage of observations that participants in single and shared bedrooms spent in social activity at each case. At Case 1, participants in single-bed rooms appeared to spend slightly less time in social activity than participants in shared bedrooms (single-bed rooms: median = 19.4%, IQR 12.2%, 46.5%, *n* = 13; shared bedrooms: median = 27.3%, IQR 25.5%, 37%, *n* = 7), which reflected the time spent alone in single and shared bedrooms at this case (see Figure S4.1). Conversely, at Case 2, participants in single-bed rooms appeared to spend more time in social activity than participants in shared bedrooms (single-bed rooms: median = 50.8%, IQR 41.1%, 62.8%, *n* = 6; shared bedrooms: median = 23.2%, IQR 22.9%, 29.8%, *n* = 10), which is somewhat unexpected since participants in single-bed rooms at this case appeared to spend more time alone than participants in shared bedrooms (see Figure S4.1).


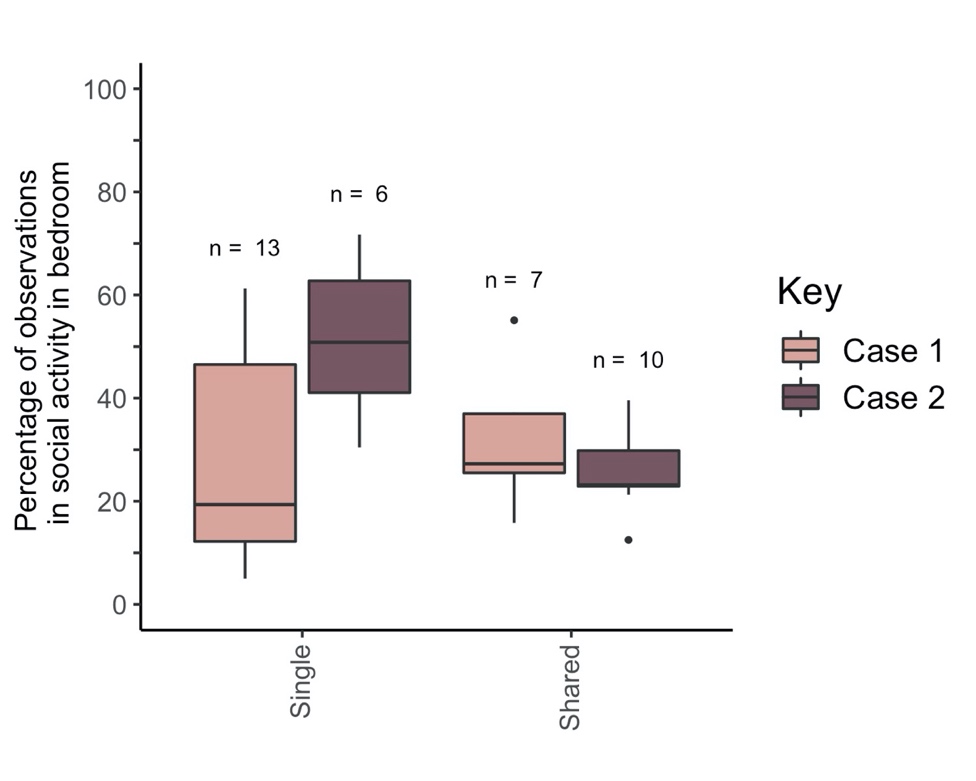

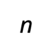

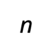

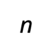

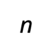


Figure S4.2. Median percentage of observations spent in social activity single and shared bedrooms by all participants at each case. The number of observations that participants were alone as a percentage of the total number of observations that they spent in their bedroom. Boxes indicate median and interquartile range. Dots represent outliers. Single = single-bed room, Shared = shared bedroom.

At Case 1, participants in single-bed rooms were socially active for 71% of the time that another person was present and participants in shared bedrooms were socially active for 31% of the time that another person was present. At Case 2, participants in single-bed rooms were socially active for 96% of the time that another person was present and participants in shared bedrooms were socially active for 29% of the time that another person was present. In other words, at both cases, the time that participants spent with others appeared to be more ‘productive’ (i.e., resulted in more social activity) in single-bed rooms than in shared bedrooms. This makes sense as other people would normally only enter a single-bed room in order to interact with the participant in some way, whereas other people may be present in a shared bedroom with no need to interact with the participant.

This effect (i.e., more social activity when other people present in single compared to shared bedrooms) appeared to be much stronger at Case 2 compared to Case 1. The single-bed rooms at Case 2 were particularly productive for social activity (i.e., social activity occurred almost the whole time that someone else was present) and the shared bedrooms were particularly unproductive (i.e., social activity occurred for a very small percentage of the time that other people were present). The difference in the ‘productivity’ of single and shared bedrooms at Case 2 may help to explain why there was more social activity in single-bed rooms compared to shared bedrooms at this case (see Figure S4.2). The beds in the shared bedrooms at Case 2 were on opposite sides of the room (rather than alongside each other) and were separated by the retractable curtains mentioned above. These design features, and possibly others, were likely introduced to the shared bedrooms to foster patient privacy but they may have had the additional effect of reducing social interaction.

*S4. 2. Further findings from the behavioural mapping regarding time alone and activity completed by participants with language and/or cognitive impairments*

At both cases, participants with language and/or cognitive impairments appeared to spend more time alone in a single-bed room than in a shared bedroom, but participants without these impairments appeared to spend a similar time alone regardless of whether they were in a single or shared bedroom. At Case 1, participants with language and/or cognitive impairments appeared to spend less time inactive overall than participants without these impairments, but the opposite was true at Case 2 (see Table S4.1). Similarly, at Case 1, participants with language and/or cognitive impairments appeared to spend more time in physical, cognitive, and social activity overall than participants without these impairments, but again the opposite was true at Case 2 (see Table S4.1). The majority of the additional physical activity undertaken by participants with language and/or cognitive impairments at Case 1 occurred in the bedroom. The additional cognitive and social activity completed by participants with language and/or cognitive impairments at Case 1 occurred in the bedroom, therapy areas, and communal areas.

Table S4.1. The percentage of observations that participants with and without language and cognitive impairments spent in physical, cognitive, and social activity at each case.

|  | | **Case 1**  ***n* = 20** | | **Case 2**  ***n* = 16** | |
| --- | --- | --- | --- | --- | --- |
| **Activity category** | | **Impairment**  ***n* = 8** | **Without**  ***n* = 12** | **Impairment**  ***n* = 12** | **Without**  ***n* = 4** |
| **Inactive, median (IQR)** | | 12.3  (2.8, 27.3) | 31.5  (19.1, 37.5) | 38.9  (18.5, 44.7) | 22.2  (12.5, 32.4) |
| **Active, median (IQR)** | |  |  |  |  |
|  | **Physical activity** | 68.3  (59.7, 80.1) | 44.4  (35.2, 51.9) | 34.3  (25.5, 41.2) | 43.5  (36.1, 50) |
|  | **Cognitive activity** | 32.1  (18.1, 37.9) | 9.3  (5.6, 16.2) | 2.78  (0, 13.9) | 3.7  (0, 9.3) |
|  | **Social activity** | 37.0  (23.6, 44.7) | 41.7  (26.4, 51.9) | 28.7  (25.1, 41.7) | 36.1  (30.6, 48.1) |

Impairment = participants with language and/or cognitive impairments; Without = participants without language and/or cognitive impairments.

IQR = interquartile range

*S4. 3. Detailed emotional well-being scores*

The median depression, anxiety, and stress scores for participants at Case 1 and Case 2 are detailed in Table S4.2.

Table S4.2. Patient reported depression, anxiety, and stress.

| **Depression, anxiety, stress categories** | | **Case 1**  ***n* = 20** | **Case 2**  ***n* = 16^a^** |
| --- | --- | --- | --- |
| **Depression scale, median (IQR) [*n*]** | | 4 (0, 15) [20] | 16 (10.5, 25.5) [14] |
|  | **Normal (score 0-9), *n* (%)** | 14 (70) | 3 (18.7) |
|  | **Mild (score 10-13), *n* (%)** | 0 (0) | 3 (18.7) |
|  | **Moderate (score 14-20), *n* (%)** | 3 (15) | 3 (18.7) |
|  | **Severe (score 21-27), *n* (%)** | 1 (5) | 2 (12.5) |
|  | **Extremely severe (score 28+), *n* (%)** | 2 (10) | 3 (18.7) |
| **Anxiety scale, median (IQR) [*n*]** | | 6 (1.5, 8) [20] | 17 (3, 27.5) [14] |
|  | **Normal (score 0-7), *n* (%)** | 14 (70) | 5 (31.3) |
|  | **Mild (score 8-9), *n* (%)** | 2 (10) | 0 (0) |
|  | **Moderate (score 10-14), *n* (%)** | 3 (15) | 1 (6.3) |
|  | **Severe (score 15-19), *n* (%)** | 0 (0) | 3 (18.7) |
|  | **Extremely severe (score 20+), *n* (%)** | 1 (5) | 5 (31.3) |
| **Stress scale, median (IQR) [*n*]** | | 8 (1.5, 16) [20] | 19 (8, 27.5) [14] |
|  | **Normal (score 0-14), *n* (%)** | 14 (70) | 5 (31.3) |
|  | **Mild (score 15-18), *n* (%)** | 4 (20) | 2 (12.5) |
|  | **Moderate (score 19-25), *n* (%)** | 0 (0) | 2 (12.5) |
|  | **Severe (score 26-33), *n* (%)** | 1 (5) | 3 (18.7) |
|  | **Extremely severe (score 34+), *n* (%)** | 1 (5) | 2 (12.5) |

Acronyms: IQR = interquartile range

^a^The Depression Anxiety Stress Scales were not completed by 2 participants at Case 2. Percentages show percent of all participants.

Table S4.3. Patient reported boredom at each case and population norms.

| **Boredom categories** | | **Case 1**  ***n* = 20** | **Case 2**  ***n* = 16** | **Population norms^a^**  ***n* = 1,691 to 1,715** |
| --- | --- | --- | --- | --- |
| **MSBS total,**  **median (IQR) [*n*]^b^**  **mean (SD)**  **z score** | | 119 (67.5, 128) [15]  97.4 (38.6)  0.0 | 127 (95.8, 149) [14]  117 (45.9)  0.5 | --  97 (38.8)  -- |
|  | **Disengagement** | 40 (29, 43) [17]  35.9 (11.9)  0.0 | 40 (27.5, 56.2) [14]  40.2 (18)  0.3 | --  35.7 (14.4)  -- |
|  | **High Arousal** | 13 (8.5, 20.5) [19]  14.5 (7.3)  -0.3 | 13 (8.3, 19.8) [14]  15.4 (9.1)  -0.1 | --  16.4 (7.5)  -- |
|  | **Inattention** | 15 (11.5, 19.8) [18]  14.9 (6.1)  0.1 | 16.5 (12.2, 22) [14]  16.1 (7.1)  0.3 | --  14.2 (6)  -- |
|  | **Low Arousal** | 18 (6.5, 20.5) [19]  15.3 (8.3)  -0.2 | 21 (16.2, 25.5) [14]  20.6 (9.5)  0.5 | --  16.6 (8.2)  -- |
|  | **Time Perception** | 18.5 (13, 23) [18]  18.7 (7.8)  0.7 | 26.5 (18, 32.8) [14]  25.1 (8.6)  1.6 | --  14.1 (7.1)  -- |

Acronyms: MSBS = Multidimensional State Boredom Scale, IQR = interquartile range.

MSBS Total minimum score = 29, maximum score = 203. Disengagement subscale minimum score = 10, maximum score = 70. High Arousal subscale minimum score = 5, maximum score = 35. Inattention subscale minimum score = 4, maximum score = 28. Low Arousal subscale minimum score = 5, maximum score = 35. Time Perception subscale minimum score = 5, maximum score = 35. Higher scores indicate more boredom.

^a^Australian population norms (non-hospitalised sample) are published by Oxtoby et al. (2016). Medians and IQRs of the population norms are not provided in this publication. The exact *n* for each subscale is not specified in the publication, but it is stated that the *n* for each subscale varied between 1,691 and 1,715.

^b^Subscales were completed by a different number of participants at each case.

Participants at both cases, and especially at Case 2, appeared to report more changes in their perception of time on the MSBS compared to the population norm. There were some features in the bedrooms at Case 2 which may have impacted patients’ time perception and could therefore explain the apparently higher score on this subscale at Case 2 compared to Case 1. For example, there were no clocks on the walls in the bedrooms at Case 2, whereas all of the bedrooms at Case 1 had clocks (most of which were set at the correct time), and the window blinds at Case 2 were much better at blocking out light, making it harder to follow the passing of time if they were closed during the day.

*S4.4. Further findings regarding emotional well-being in single and shared bedrooms*

Figure S4.3 shows the median depression, anxiety, and stress scores for participants in single and shared bedrooms at each case. At Case 1, participants in single-bed rooms appeared to experience similar levels of depression, anxiety, and stress as participants in shared bedrooms. At Case 2, participants in single-bed rooms appeared to experience similar levels of anxiety as participants in shared bedrooms, but higher levels of depression and stress. Participants in both single and shared bedrooms at Case 2 appeared to experience more severe levels of depression, anxiety, and stress compared to participants in either room type at Case 1, that is, depression, anxiety, and stress appeared to vary more by case than by number of beds in the room (see Figure S4.3). This suggests that other factors besides the number of beds in the room played a more important role in participants’ mood. These other factors may have been internal participant competencies (such as the demographic and clinical characteristics which differed between cases) and/or external factors in the social and physical environment such as roommates’ impairments, type of barriers/curtains between beds, position of bedroom relative to communal hubs, etc.


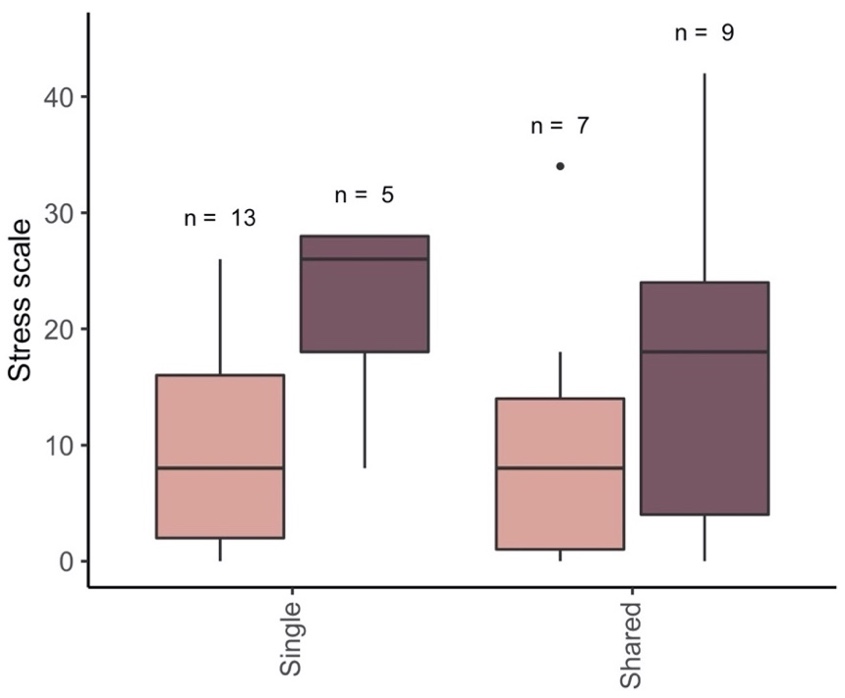

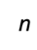

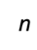

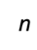

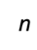

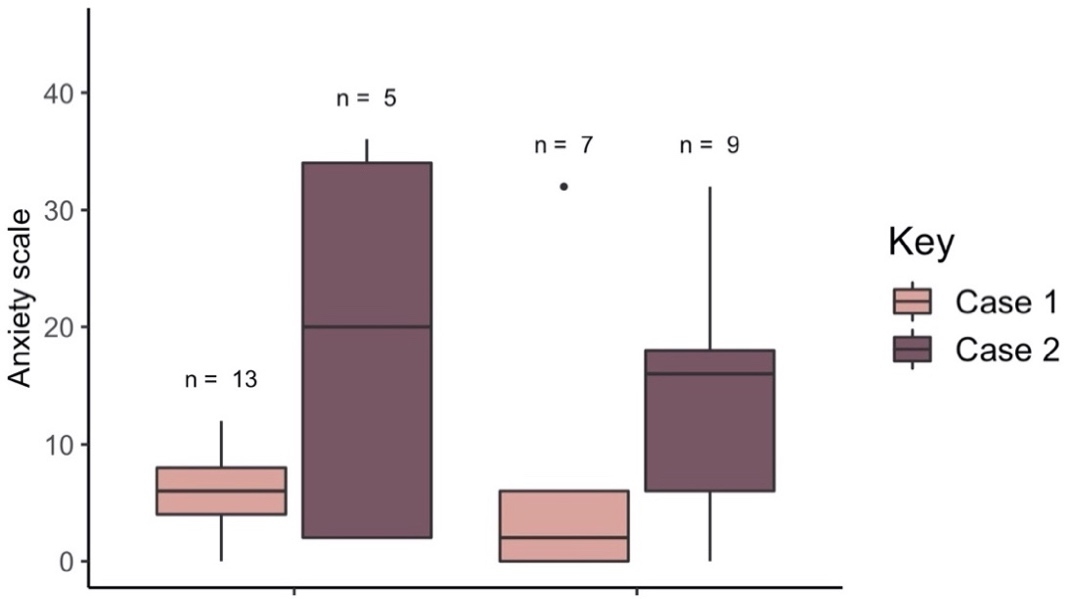

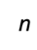

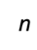

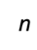

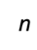

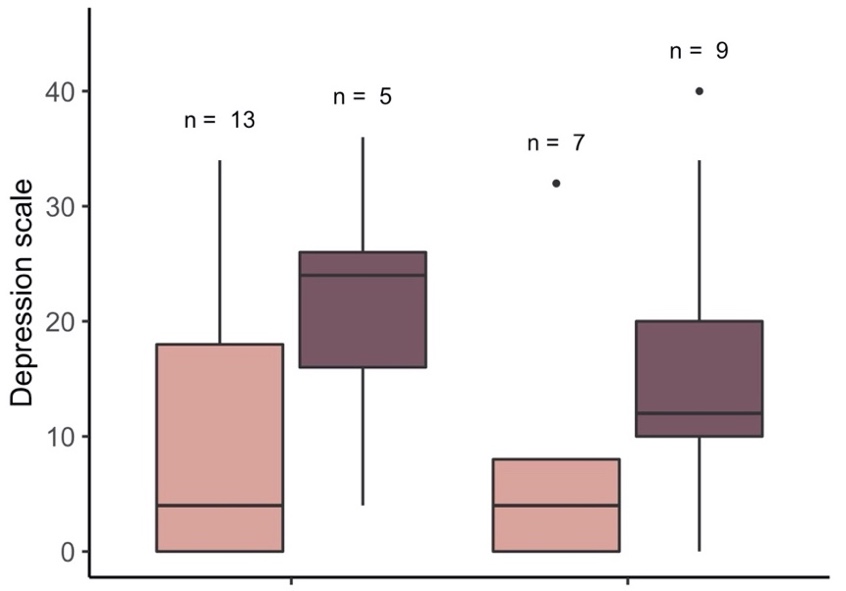

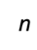

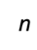

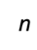

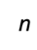


Figure S4.3. Median depression, anxiety, and stress reported by participants in single and shared bedrooms at each case. Higher scores on the Depression, Anxiety, Stress Scales (DASS) indicate higher levels of depression, anxiety, and stress. Boxes indicate median and interquartile range. Dots represent outliers. Single = single-bed room, Shared = shared bedroom.

Figure S4.4 shows the median boredom scores for participants in single and shared bedrooms at each case. At both cases, participants in single-bed rooms appeared to experience more boredom than participants in shared bedrooms. At Case 2, the mean MSBS score was higher than the population mean for participants in single-bed room but within half a standard deviation of the population mean for participants in a shared bedroom (single-bed room: mean = 139, *SD* = 25.2, *z* score = 1.1; shared bedroom: mean = 105, *SD* = 51.5, *z* score = 0.2); while at Case 1, the mean score for participants in a single-bed room was similar to the population mean and the score for participants in a shared bedroom was lower than the population mean (single-bed room: mean = 109, *SD* = 34, *z* score = 0.3; shared bedroom: mean = 74.6, *SD* = 40.5, *z* score = -0.6). Participants were more alone in single-bed rooms at both cases (see Figure S4.1), which may have contributed to their boredom.


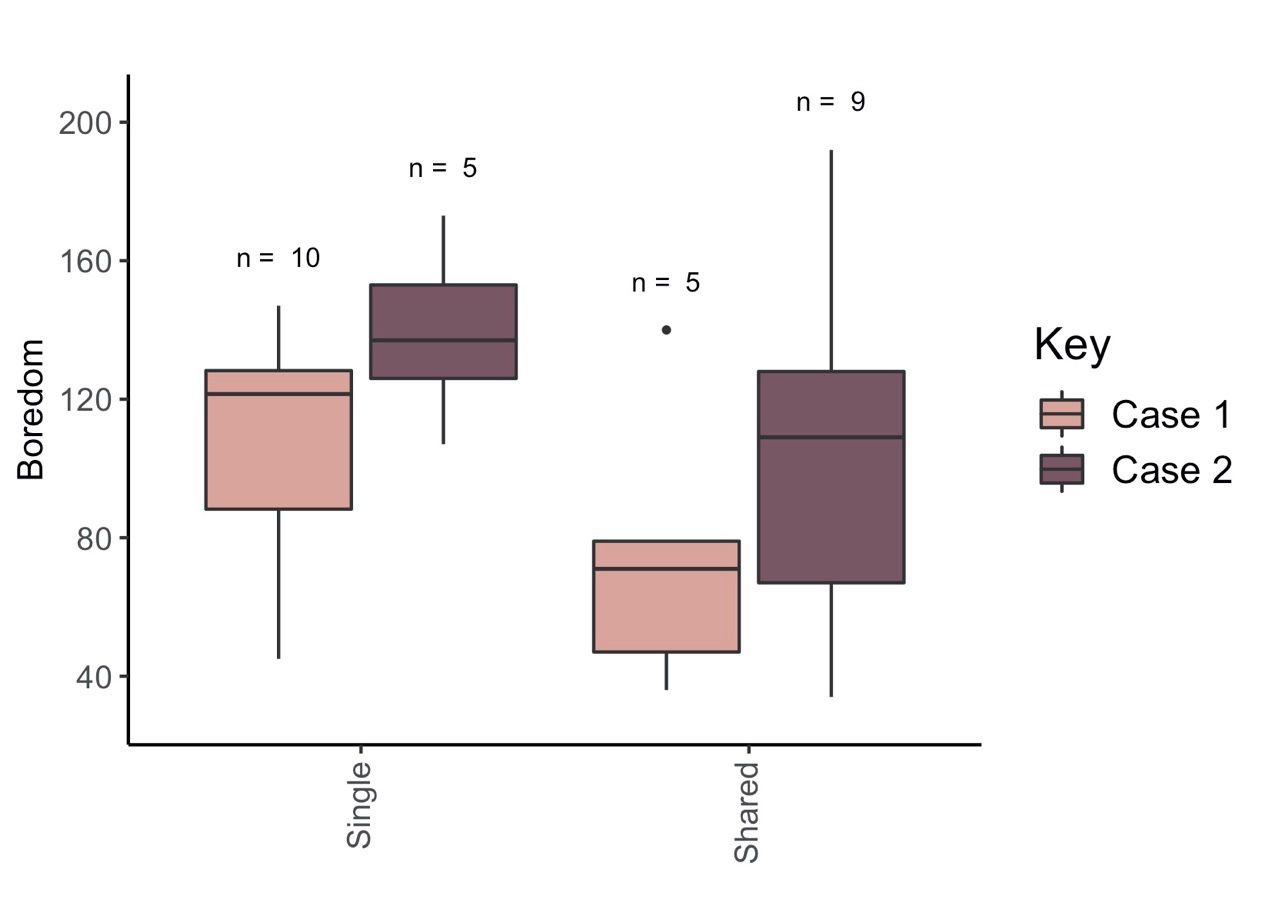

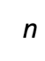

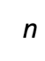

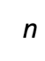

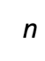


Figure S4.4. Median boredom reported by participants in single and shared bedrooms at each case. Higher scores on the Multidimensional State Boredom Scale (MSBS) indicate higher levels of boredom. Boxes indicate median and interquartile range. Dots represent outliers. Single = single-bed room, Shared = shared bedroom.

Figure S4.5 shows the VAS scores for participants in single and shared bedrooms at both cases. Participants in single-bed rooms at Case 2 appeared to find the environment more motivating than participants in shared bedrooms at this case, but participants in single and shared bedrooms at both cases varied widely in how motivating they found the environment (see medians and IQRs in Figure S4.5). This wide spread of responses suggests that motivation, like mood, may have been driven more by other factors than by the number of beds in the room.


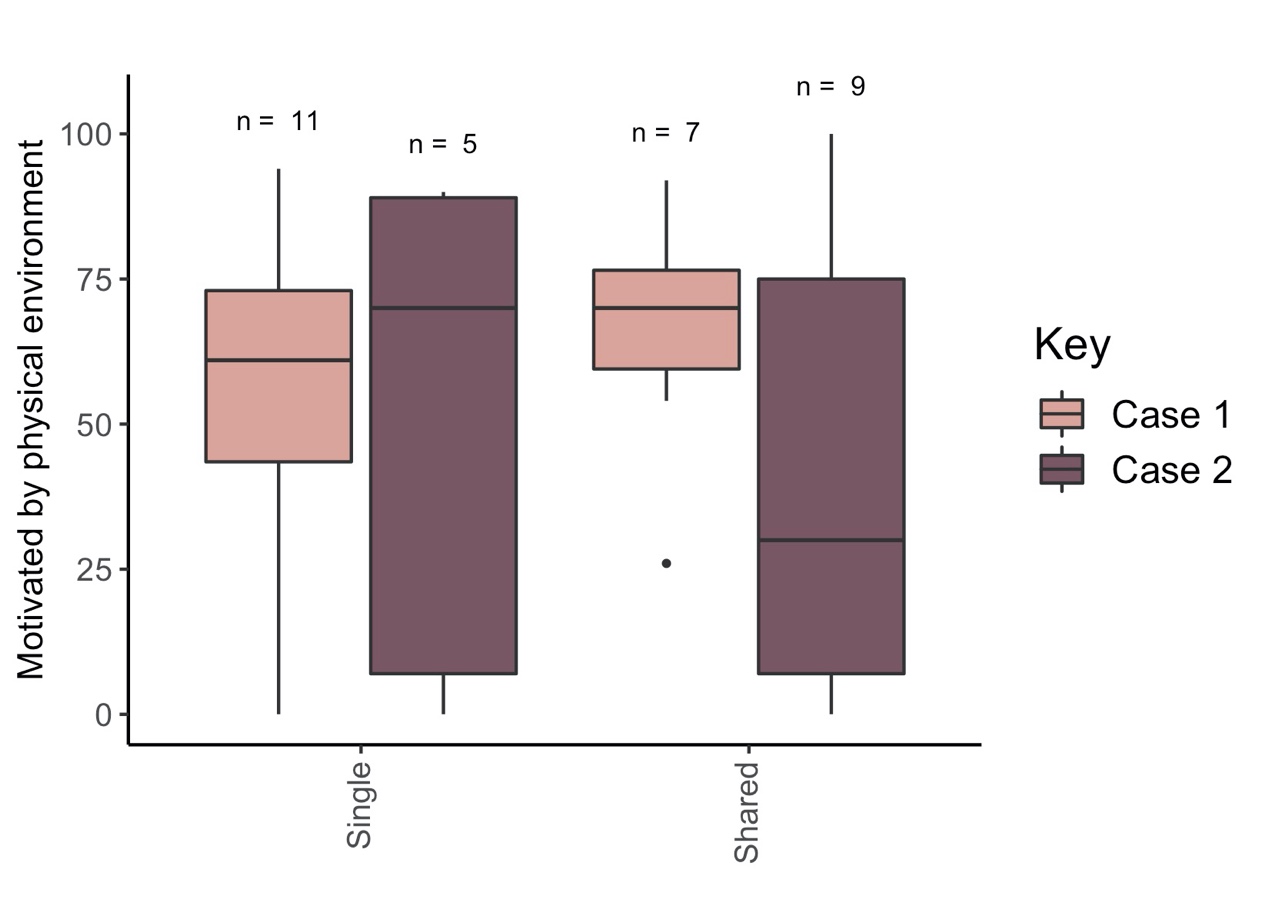

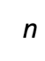

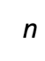

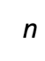

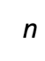


Figure S4.5. The median extent to which participants in single and shared bedrooms at each case found the physical environment to be motivating for rehabilitation. The extent to which the physical environment was motivating was measured using a Visual Analogue Scale where a score of 100 indicated that the physical environment was strongly motivating and a score of 0 indicated that it was strongly demotivating. Boxes indicate median and interquartile range. Dots represent outliers. Single = single-bed room, Shared = shared bedroom.
